# Exploring ethical considerations in medical research: Harnessing pre-generated transformers for AI-powered ethics discussions

**DOI:** 10.1371/journal.pone.0311148

**Published:** 2025-02-03

**Authors:** Takuya Mori, Takuya Watanabe, Shinji Kosugi

**Affiliations:** 1 Department of Ethics Support, Kyoto University Hospital, Kyoto, Japan; 2 Department of Medical Genetics and Medical Ethics, Graduate School of Medicine, Kyoto University, Kyoto, Japan; Central Queensland University, AUSTRALIA

## Abstract

**Introduction:**

In medical research involving human subjects, ethical review is essential to protect individuals. However, concerns have been raised about variations in ethical review opinions and a decline in review quality. Adequately protecting human subjects requires multifaceted opinions from ethics committee members. Despite the need to increase the number of committee members, resources are limited. To address these challenges, we explored the use of a generative pre- learning transformer, an interactive artificial intelligence (AI) tool, to discuss ethical issues in medical research.

**Methods:**

The generation AI used in the research used ChatGPT3.5, which has learned ethical guidelines from various countries worldwide. We requested the generative AI to provide insights on ethical considerations for virtual research involving individuals. The obtained answers were documented and verified by experts.

**Results:**

The AI successfully highlighted considerations for informed consent regarding individuals with dementia and mental illness, as well as concerns about invasiveness in research. It also raised points about potential side effects of off-label drug use. However, it could not offer specific measures for psychological considerations or broader ethical issues, providing limited ethical insights. This limitation may be attributed to biased opinions resulting from machine learning optimization, preventing comprehensive identification of certain ethical issues.

**Conclusion:**

Although the validity of ethical opinions generated by the generative AI requires further examination, our findings suggest that this technology could be employed to prompt reviews and re-evaluate ethical concerns arising in research.

## Introduction

Research involving human subjects must undergo ethical review before initiation to protect their welfare and rights. Each country has established guidelines, regulations, and ethics review committees to ensure compliance [1, 2]. In the medical field, international ethical guidelines such as the Belmont Report [3] and the Declaration of Helsinki [4] provide foundational concepts. These related ethical guidelines and regulations are important from the viewpoint of international and universal review, but the most crucial aspect is the quality of review by ethical review committees. In an effort to ensure the quality of ethics committee reviews, the World Health Organization and the Council for International Organizations of Medical Sciences have issued guidelines on review quality and practices [5]. In recent years, there have been accreditation systems for ethics committees, wherein a third-party organization evaluates their quality. For instance, in the United States, the Association for the Accreditation of Human Research Protection Programs (AAHRPP) oversees the Human Research Protection Programs [6]. In the Western Asia-Pacific region, the Forum for Ethical Review Committees in the Asian and Western Pacific Region plays a similar role [7]. In Japan, the Ministry of Health, Labor, and Welfare has introduced a certification system without exception [8]. Establishing an organizational system for ethical review and receiving certification from an external organization is highly beneficial for ensuring the protection of research subjects. Meanwhile, to address the quality issues of actual ethics committees, it is considered most important for the members of each committee to “provide a comprehensive opinion of ethical considerations for the protection of human subjects” [9–11]. This stems from a recent survey conducted by AAHRPP accrediting bodies, which raises questions about whether improvements to the system alone can sufficiently address the quality of the ethics review [12]. For instance, even when an ethical review takes place, it remains a formal process, and the absence of ethical opinions regarding human protection is a significant concern. Additionally, in some countries, including Japan, collaborative research involving multiple institutions is typically subjected to review by a single ethics committee [13]. Although a single ethics review streamlines procedures and consolidates opinions, ongoing debates question the quality of such reviews [11, 14]. To enhance opinions on ethics reviews, efforts are being made to elevate the quality of reviews by providing advanced education to ethics committee members and fostering opinion exchanges among committees at the national level. Nevertheless, in the current Japanese context, there are suspicions that the quality of reviews is declining in the humanities and social sciences due to disparities in opinions and review resources resulting from the excessive establishment of ethics committees, and the medical field is no exception [15]. Addressing this disparity in opinions among ethics committees is crucial for unifying the quality of ethical reviews. Therefore, as a pioneering effort to extract specific opinions, we conceived the idea of utilizing natural language processing in artificial intelligence (AI) to extract ethical opinions on research issues [16]. An AI capable of detecting such errors could be highly valuable, particularly in thoroughly discussing ethical considerations within a research protocol. Generative Pre-trained Transformer (ChatGPT), an interactive AI that underwent extensive language learning, has received attention in recent years. ChatGPT, developed by OpenAI, admits mistakes based on the results of a huge database and supervised machine learning. Although its accuracy is continuously reviewed, it indicates the potential to challenge incorrect assumptions [17]. Additionally, it can follow the content of a previous dialogue, answering questions without the need for repeated back-and-forth exchanges within a single dialogue [18]. Released in November 2022, ChatGPT’s AI dialogue system gained global recognition and was quickly employed in medical research. It facilitated professional discussions with experts on the biological role of computational systems in stem cell research [19]. ChatGPT also explored the use of rapamycin, a drug preventing organ transplant rejection, showcasing substantial expertise in understanding its effects and side effects and responding appropriately [20]. These observations suggest the potential use of generative AI in discussing ethical considerations in research. However, there are currently no reports that have queried ChatGPT about the ethical viewpoint of research and disclosed the results. Consequently, it remains intriguing to explore whether opinions from the standpoint of research ethics can be extracted.

To this end, the present study aimed to input summaries of various medical studies into ChatGPT and examine whether interactive AI can identify ethical issues in research.

## Methods

In this study, we used ChatGPT (January 30 Version, OpenAI LP, https://chat.openai.com/chat) [21]. We chose this version as it was the latest available when the research commenced and was easily accessible with a simple account registration, making it widely applicable. Additionally, we select ChatGPT over other generative AI tools due to its extensive testing and numerous reports. We structured questions simulating research scenarios with ethical considerations and directed them to ChatGPT, adopting its responses as our results. When designing a research scenario, we encountered a processing limit of approximately 2000 words with ChatGPT-3.5, making it challenging to process documents of the same length as our actual research plan. Therefore, we decided to create a concise English version of ≤100 words, considering ChatGPT’s performance degradation with increasing word count and the need for Japanese language support. Consequently, for this study, we used ChatGPT for interactive exploration of ethical considerations in hypothetical research queries instead of adapting lengthy documents such as research plans. The questions asked to ChatGPT were divided into five main categories: “Q1. Learning status of ethical principles in medical research ethics by ChatGPT”; “Q2. Opinions regarding ethical considerations for patients with dementia and depression”; “Q3. Opinions regarding ethical considerations for invasiveness and risks to subjects”; “Q4. Opinions regarding ethical considerations for the requirement of informed consent”; “Q5. Regarding research ethics judgment by ChatGPT.” Detailed contents are provided below (questions entered into ChatGPT). To ensure the reproducibility of ChatGPT’s responses, we repeated the same questions multiple times and confirmed consistency in the answers. As this study is exploratory in nature and focuses on the content rather than external evaluation indicators for assessing the ethics and accuracy of ChatGPT responses, the results output from ChatGPT are tabulated. Two research ethics experts then engaged in open-ended discussions about the content, comparing and discussing it in light of previously published studies. These discussions included assessing whether ChatGPT responses were comparable to the human subject protection content reported in previously published research ethics papers. Furthermore, because this study did not involve personal information or human subjects, it falls outside the scope of Japan’s ethical guidelines. Consequently, the Kyoto University Graduate School and Faculty of Medicine Ethics Committee determined that ethical review was unnecessary.

### Questions entered into ChatGPT

**Q1: Learning status of ethical principles in medical research ethics by ChatGPT**. The following questions were asked to verify the understanding of fundamental ethical principles in medical research by ChatGPT.

Q1-1: Is ChatGPT learning the Declaration of Helsinki?

Q1-2: Is ChatGTP learning the Belmont Report?

Q1-3: Is ChatGPT learning the ethical guidelines for Medical and Health Research Involving Human Subjects?

**Q2: Opinions regarding ethical considerations for patients with dementia and depression**. The following questions were asked regarding virtual research for patients with dementia and psychiatric diseases, who require special consideration when making decisions before participating in research.

Q2-1: In a study of 50 dementia patients, we will analyze specific proteins in the blood of cognitive function tests and blood samples, and conduct research to confirm the correlation between cognitive function and specific proteins. What are the ethical considerations required in this case?

Q2-2: In a study of 500 depressed patients, classifying their mental states using a test of more than 50 questions that takes 1 to 2 hours to answer, and exploring the correlation between their mental states and brain function measured by MRI. What are the ethical considerations required in this case?

**Q3: Opinions regarding ethical considerations for invasiveness and risks to subjects**. The following questions were asked regarding hypothetical studies involving invasiveness and risks, including lumbar punctures and off-label use of anticancer drugs.

Q3-1: In a study of 100 dementia patients, we will conduct a lumbar puncture, analyze proteins in the cerebrospinal fluid, and investigate the relationship with the rate of decline in cognitive function. What are the ethical considerations required in this case?

Q3-2: In pancreatic cancer patients, off-label use of anticancer drugs that are usually used for skin cancer, and research to confirm the patient’s survival prognosis and pancreatic cancer disease progression. What are the ethical considerations required in this case?

**Q4: Opinions regarding ethical considerations for the requirement of informed consent.** The following questions were asked concerning doubts and risks associated with specific content in the informed consent process for invasive virtual research involving patients with dementia.

Q4-1: In a study of 100 dementia patients, we will conduct a lumbar puncture, analyze proteins in the cerebrospinal fluid, and investigate the relationship with the rate of decline in cognitive function. What informed consent is required before starting research?

Q4-2: Is it necessary to consider dementia when giving explanations?

Q4-3: What are the possible risks?

**Q5: Regarding research ethics judgment by ChatGPT**. We asked the following question about ChatGPT’s ability to make ethical decisions regarding research subjects.

Q5-1: Can ChatGPT make ethical judgments about research?

## Results

ChatGPT provided relevant answers to all 11 questions (Q1-1 to Q5-1). In response to Q1, ChatGPT indicated that it had studied the Declaration of Helsinki, the Belmont Report, and Japan’s unique ethical guidelines for medical research involving humans. It asserted its ability to propose ethical considerations based on these guidelines (Table 1). Furthermore, when interpreting Japan’s unique ethical guidelines for medical research involving human subjects, ChatGPT demonstrated an understanding that these guidelines encompass the Belmont Report, Declaration of Helsinki, and Council for International Organizations of Medical Sciences (Table 1).

**Table 1:** Learning status of ethical principles in medical research ethics by ChatGPT.

Number	Questions from researchers to ChatGPT	ChatGPT Answer
1–1	**Is ChatGPT learning the Declaration of Helsinki?**	Yes, I have learned about the Declaration of Helsinki. The Declaration of Helsinki is an international ethical guideline for medical research involving human subjects. It was first adopted in 1964 and has been revised several times, most recently in 2013. The Declaration sets out principles for ethical research, including informed consent, respect for privacy and confidentiality, and protection of vulnerable groups. I am familiar with the content of the Declaration of Helsinki and can answer questions related to the ethical conduct of medical research involving human subjects based on its principles.
1–2	**Is ChatGTP learning the Belmont Report?**	Yes, I have learned about the Belmont Report. The Belmont Report is a seminal document in the field of bioethics, published in 1979 by the National Commission for the Protection of Human Subjects of Biomedical and Behavioral Research in the United States. The report outlines three basic ethical principles that should guide research involving human subjects: respect for persons, beneficence, and justice. It also discusses practical applications of these principles in research design and implementation, and provides a framework for the ethical conduct of research. I am familiar with the content of the Belmont Report and can answer questions related to the ethical conduct of research involving human subjects based on its principles.
1–3	**Is ChatGPT learning the ethical guidelines for Medical and Health Research Involving Human Subjects?**	Yes, I have learned about the ethical guidelines for medical and health research involving human subjects. These guidelines include various documents such as the Declaration of Helsinki, the Belmont Report, and the International Ethical Guidelines for Health-related Research Involving Humans published by the Council for International Organizations of Medical Sciences (CIOMS). These guidelines provide principles for the ethical conduct of research involving human subjects, including informed consent, respect for participants’ autonomy and privacy, protection of vulnerable populations, and fair distribution of the benefits and burdens of research. I am familiar with these ethical guidelines and can provide information and answers to questions related to the ethical conduct of research involving human subjects based on these principles.

In response to Q2-1 and Q2-2, ChatGPT emphasized the importance of informed consent, confidentiality and privacy, risk minimization, benefits, justice, deception, and data management in research involving patients with dementia and depression (Table 2).

**Table 2:** Opinions regarding ethical considerations for patients with dementia and depression.

Number	Questions from researchers to ChatGPT	ChatGPT Answer
2–1	A study of 50 dementia patients, we will analyze specific proteins in the blood of cognitive function tests and blood samples, and conduct research to confirm the correlation between cognitive function and specific proteins. **What are the ethical considerations required in this case?**	In conducting research involving human subjects, it’s crucial to consider ethical principles to ensure the safety, welfare, and rights of participants. Here are some of the ethical considerations for the study you described: Informed consent: Participants must be informed about the purpose, procedure, risks, and benefits of the study, and must voluntarily agree to participate. They must also have the right to withdraw from the study at any time without any negative consequences. Confidentiality and privacy: Participants’ personal information and test results must be kept confidential and secure, and appropriate measures should be taken to protect their privacy. Risk minimization: The study must be designed and conducted in a way that minimizes harm and potential risks to participants. For example, blood sampling should be done using appropriate techniques to minimize the risk of infection. Beneficence: The study should aim to maximize the potential benefits to participants and society while minimizing harm. For example, the results of the study could help improve the diagnosis and treatment of dementia. Justice: Participants must be selected fairly and equitably, and the benefits and burdens of the study must be distributed fairly. For example, researchers must avoid recruiting only participants from vulnerable populations who may be less able to give informed consent or understand the risks involved. Deception: Researchers must not deceive participants about the purpose or nature of the study. Use of placebo: If a placebo is used in the study, participants must be informed about this and their consent must be obtained. Data management: The data collected in the study must be handled, stored, and analyzed in a secure and responsible manner. It’s important to note that these ethical considerations are not exhaustive and may vary based on the specific circumstances of the study. Researchers should consult relevant ethical guidelines and obtain appropriate ethical approval before conducting the study.
2–2	A study of 500 depressed patients, classifying their mental states using a test of more than 50 questions that takes 1 to 2 hours to answer, and exploring the correlation between their mental states and brain function measured by MRI. **What are the ethical considerations required in this case?**	Conducting research involving human subjects, particularly those with mental health conditions, requires careful consideration of ethical principles to ensure the safety, welfare, and rights of participants. Here are some of the ethical considerations for the study you described: Informed consent: Participants must be informed about the purpose, procedure, risks, and benefits of the study, and must voluntarily agree to participate. They must also have the right to withdraw from the study at any time without any negative consequences. Confidentiality and privacy: Participants’ personal information and test results must be kept confidential and secure, and appropriate measures should be taken to protect their privacy. Risk minimization: The study must be designed and conducted in a way that minimizes harm and potential risks to participants. For example, the use of MRI should be justified and the potential risks of exposure to magnetic fields must be minimized. Beneficence: The study should aim to maximize the potential benefits to participants and society while minimizing harm. For example, the results of the study could help improve the understanding and treatment of depression. Justice: Participants must be selected fairly and equitably, and the benefits and burdens of the study must be distributed fairly. For example, researchers must avoid recruiting only participants from vulnerable populations who may be less able to give informed consent or understand the risks involved. Deception: Researchers must not deceive participants about the purpose or nature of the study. Mental health protection: Participants with depression may be vulnerable and susceptible to harm, and the study must be designed to minimize the potential harm to their mental health. For example, participants must be screened to ensure they are able to participate in the study without significant harm, and appropriate support must be available to them during and after the study. Data management: The data collected in the study must be handled, stored, and analyzed in a secure and responsible manner. It’s important to note that these ethical considerations are not exhaustive and may vary based on the specific circumstances of the study. Researchers should consult relevant ethical guidelines and obtain appropriate ethical approval before conducting the study.

Additionally, ChatGPT emphasized the importance of avoiding the exclusive recruitment of participants from vulnerable populations in studies involving individuals with dementia, especially those who may have difficulty comprehending the associated risks (Table 2). In the context of research on patients with depression, ChatGPT recommended measures for mental health protection, screening tests during the informed consent process, and underscored the necessity for suitable support both during and after the study (Table 2).

In response to Q3-1, ChatGPT highlighted the importance of being attentive to the vulnerability of patients with dementia during lumbar puncture procedures. Furthermore, it emphasized the need to establish a suitable medical system to mitigate potential complications, such as headaches and infections (Table 3). Regarding Q3-2, risk minimization was addressed, with ChatGPT stating that the off-label use of anticancer drugs should be implemented carefully and that a monitoring system for side effects should be established (Table 3).

**Table 3:** Opinions regarding ethical considerations for invasiveness and risks to subjects.

Number	Questions from researchers to ChatGPT	ChatGPT Answer
3–1	A study of 100 dementia patients, we will conduct a lumbar puncture, analyze proteins in the cerebrospinal fluid, and investigate the relationship with the rate of decline in cognitive function. What are the ethical considerations required in this case?	Conducting research involving human subjects, particularly those with dementia, requires careful consideration of ethical principles to ensure the safety, welfare, and rights of participants. Here are some of the ethical considerations for the study you described: Informed consent: Participants must be informed about the purpose, procedure, risks, and benefits of the study, and must voluntarily agree to participate. They must also have the right to withdraw from the study at any time without any negative consequences. Confidentiality and privacy: Participants’ personal information and test results must be kept confidential and secure, and appropriate measures should be taken to protect their privacy. Risk minimization: The study must be designed and conducted in a way that minimizes harm and potential risks to participants. For example, the lumbar puncture procedure should be done using appropriate techniques to minimize the risk of complications, such as headache or infection. Beneficence: The study should aim to maximize the potential benefits to participants and society while minimizing harm. For example, the results of the study could help improve the understanding of dementia and the development of new treatments. Justice: Participants must be selected fairly and equitably, and the benefits and burdens of the study must be distributed fairly. For example, researchers must avoid recruiting only participants from vulnerable populations who may be less able to give informed consent or understand the risks involved. Deception: Researchers must not deceive participants about the purpose or nature of the study. Vulnerability of dementia patients: Participants with dementia may be vulnerable and susceptible to harm, and the study must be designed to minimize the potential harm to their health and well-being. For example, participants must be screened to ensure they are able to participate in the study without significant harm, and appropriate support must be available to them during and after the study. Data management: The data collected in the study must be handled, stored, and analyzed in a secure and responsible manner. It’s important to note that these ethical considerations are not exhaustive and may vary based on the specific circumstances of the study. Researchers should consult relevant ethical guidelines and obtain appropriate ethical approval before conducting the study.
3–2	In pancreatic cancer patients, off-label use of anticancer drugs that are usually used for skin cancer, and research to confirm the patient’s survival prognosis and pancreatic cancer disease progression. What are the ethical considerations required in this case?	In conducting research involving human subjects, particularly those with pancreatic cancer, it’s important to consider ethical principles to ensure the safety, welfare, and rights of participants. Here are some of the ethical considerations for the study you described: Informed consent: Participants must be informed about the purpose, procedure, risks, and benefits of the study, and must voluntarily agree to participate. They must also have the right to withdraw from the study at any time without any negative consequences. Confidentiality and privacy: Participants’ personal information and test results must be kept confidential and secure, and appropriate measures should be taken to protect their privacy. Risk minimization: The study must be designed and conducted in a way that minimizes harm and potential risks to participants. For example, the off-label use of anticancer drugs should be done with caution, and participants must be monitored for any adverse effects. Beneficence: The study should aim to maximize the potential benefits to participants and society while minimizing harm. For example, the results of the study could help improve the understanding of pancreatic cancer and the development of new treatments. Justice: Participants must be selected fairly and equitably, and the benefits and burdens of the study must be distributed fairly. For example, researchers must avoid recruiting only participants from vulnerable populations who may be less able to give informed consent or understand the risks involved. Deception: Researchers must not deceive participants about the purpose or nature of the study. Vulnerability of patients: Participants with pancreatic cancer may be vulnerable and susceptible to harm, and the study must be designed to minimize the potential harm to their health and well-being. For example, participants must be screened to ensure they are able to participate in the study without significant harm, and appropriate support must be available to them during and after the study. Data management: The data collected in the study must be handled, stored, and analyzed in a secure and responsible manner. It’s important to note that these ethical considerations are not exhaustive and may vary based on the specific circumstances of the study. Researchers should consult relevant ethical guidelines and obtain appropriate ethical approval before conducting the study.

In response to Q4-1, as a standard practice, written consent was recommended, and ChatGPT provided responses to 14 items in line with international ethical principles regarding precautions for informed consent explanations (Table 4). For Q4-2, ChatGPT emphasized the importance of considering the subject’s comprehension and, in certain situations, obtaining informed consent from an authorized legal representative or family member (Table 4). Addressing Q4-3, ChatGPT explicitly listed headache, infection, bleeding, and allergic reactions as the most prevalent risks. Moreover, acknowledging the potential for other complications, it underscored the significance of informed consent (Table 4).

**Table 4:** Opinion on ethical considerations for the requirement of informed consent.

Number	Questions from researchers to ChatGPT	ChatGPT Answer
4–1	A study of 100 dementia patients, we will conduct a lumbar puncture, analyze proteins in the cerebrospinal fluid, and investigate the relationship with the rate of decline in cognitive function. **What informed consent is required before starting research?**	Before starting the research, you will need to obtain informed consent from the participants or their authorized representatives. Informed consent is a voluntary agreement by a competent individual to participate in research after receiving information about the nature of the research, its risks and benefits, and their rights as research participants. The informed consent process for this study should include the following elements: Explanation of the purpose, procedures, and risks of the lumbar puncture and protein analysis Description of the potential benefits and harms of the study Explanation that participation is voluntary and that the participant can withdraw at any time Description of how confidentiality will be maintained and who will have access to the participant’s information Disclosure of any financial or personal conflicts of interest of the researcher Explanation of any compensation for participation, if applicable Opportunity for the participant to ask questions and to receive additional information about the study. It is important to ensure that the participants or their authorized representatives fully understand the information presented and provide their consent voluntarily without coercion or undue influence. The consent process should also be documented by obtaining a signed informed consent form or another form of documented consent.
4–2	**Is it necessary to consider dementia when giving explanations?**	Yes, it is important to consider the patients’ cognitive status and level of understanding when providing explanations about the research and obtaining informed consent, especially for patients with dementia. Patients with dementia may have difficulty understanding complex information or retaining new information due to memory impairment and cognitive decline. As such, it is important to use plain language and simple terms to explain the purpose, procedures, risks, and benefits of the study in a clear and concise manner. You may also need to repeat the information or break it down into smaller parts to ensure the patient understands it. If the patient is unable to give informed consent due to their cognitive impairment, their authorized representative (such as a family member or legal guardian) may provide informed consent on their behalf. In this case, it is important to ensure that the representative understands the information and is acting in the best interest of the patient. In summary, considering the patients’ cognitive status and level of understanding is crucial to obtain valid informed consent and ensure that patients with dementia are not taken advantage of in research studies.
4–3	**What are the possible risks?**	In this specific study, the lumbar puncture procedure carries some risks, although they are generally rare. Some possible risks associated with lumbar puncture include: Headache: This is the most common complication, affecting up to 40% of patients. It usually occurs within the first few hours after the procedure and can last for several days. Infection: Although rare, infection of the cerebrospinal fluid or surrounding tissues can occur, which may require further treatment. Bleeding: Rarely, the needle may cause bleeding within the spinal canal, which may result in nerve damage, paralysis, or even death. Allergic reaction: Patients may have an allergic reaction to the anesthetic used during the procedure or to the contrast dye, if used. Other risks: There may be other, less common risks associated with lumbar puncture, such as back pain or discomfort, loss of cerebrospinal fluid, and injury to other structures within the spinal canal. It is important to thoroughly explain these potential risks to the patients or their authorized representatives as part of the informed consent process. This allows them to make an informed decision about whether to participate in the study or not. Additionally, it is important to monitor the patients carefully after the procedure to detect and manage any potential complications that may arise.

In response to Q5, ChatGPT was found to understand relevant laws and regulations related to research ethics and ethical review, allowing it to make proposals in accordance with research ethics principles; however, ChatGPT could not make judgments (Table 5).

**Table 5:** Regarding research ethics judgment by ChatGPT.

**Number**	**Questions from researchers to ChatGPT**	**ChatGPT Answer**
5–1	Can ChatGPT make ethical judgments about research?	As an AI language model, I can provide information and guidance on ethical principles and considerations in research, but I cannot make ethical judgments about research. Ethical judgments often require a nuanced understanding of the specific context, and are typically made by individuals or groups with specialized training and expertise in the relevant fields of study. Ethical considerations in research often involve complex and multifaceted issues, including balancing potential benefits and risks, ensuring participant autonomy and informed consent, minimizing harm, maintaining confidentiality and privacy, and ensuring fairness and justice. It is important for researchers to carefully consider these issues and seek the guidance of institutional review boards (IRBs), ethics committees, or other relevant authorities to ensure that their research is conducted in an ethically responsible manner. As an AI language model, my role is to provide information and support to individuals seeking guidance on ethical issues in research. Ultimately, ethical judgments must be made by those with the necessary expertise, training, and authority to ensure that research is conducted in an ethical and responsible manner.

## Discussion

In this study, we verified the learning status of ChatGPT regarding research ethics and tasked it with addressing questions about ethical considerations related to subject protection. Initially, when inquiring about ChatGPT’s understanding of the Belmont Report and the Declaration of Helsinki, it accurately provided the adoption years and history of revisions. Moreover, ChatGPT demonstrated familiarity with informed consent, privacy protection, protection of vulnerable groups, and Japan’s unique ethical guidelines. Although ChatGPT could answer questions based on these principles without error, it remains unclear whether it can apply ethical principles to diverse research scenarios and respond flexibly to subject protection opinions. Subsequently, when asked about ethical considerations for an observational study involving 50 patients with dementia, ChatGPT emphasized the importance of informed consent, confidentiality and privacy, risk minimization, beneficence, justice, deception, and data management. It also highlighted the need to minimize infection risks from blood collection and stressed the voluntary participation of vulnerable subjects. These responses were based on formal ethical principles, outlining essential elements for any research [3, 4]. Some answers delved into the risks of blood sampling and the imperative to maximize the benefits of diagnosing and treating patients with dementia. Notably, the varying levels of detail and abstraction in ChatGPT’s responses may result from machine learning, possibly reducing opinions to the minimum required [22]. Another study reported a phenomenon wherein ChatGPT responses were consistently repeated in a fixed form. It is plausible that it provided answers to questions related to this research with a certain degree of prepared responses [18]. Next, we assessed the responses to a questionnaire comprising more than 50 questions directed at 500 patients with depression, along with ChatGPT’s perspectives on research involving the analysis of brain function through MRI. An explicit viewpoint emerged, emphasizing the vulnerability of participants with depression and the necessity for studies to minimize potential harm to their mental health. Consequently, there was an identified need for participants to undergo screening, ensuring their ability to participate without causing serious harm, with adequate support during and after the research. Although there was no commentary on the sample size, we interpret these responses as establishing a link between the mental burden from the questionnaire and depression. Reports on ethical considerations for treating depression highlight the importance of assessing and supporting potential problems on a case-by-case basis [23, 24]. The significance of informed consent and confidentiality in research on mental disorders has been previously reported [25]. The role of informed consent in addressing psychiatric issues has also been a subject of questioning, particularly in the context of editorial responsibilities for psychiatric journals [26]. These findings align with the responses provided by ChatGPT. Furthermore, ChatGPT addressed considerations for a study involving lumbar puncture for dementia patients, expressing concern about the potential risks of headaches and infections. It emphasized the necessity of establishing a reliable medical system to mitigate these risks, addressing typical complications associated with lumbar puncture [27, 28], and we believe that these responses demonstrate a professional understanding of the risks involved. Additionally, in the virtual study on the off-label use of anticancer drugs in patients with pancreatic cancer, ChatGPT asserted the importance of monitoring side effects and striving to reduce associated risks. In essence, opinions focused on the establishment of a system capable of handling side effects and ensuring the thoroughness of free consent regarding the off-label use of pharmaceuticals. Interestingly, ChatGPT provided insights into the immunosuppressive drug rapamycin and discussed its range of indications and concerns in vivo when used off-label [20]. By subdividing questions within ChatGPT, it could offer expert insights into the drug’s side effects. Next, when inquired about the informed consent required for research on patients with dementia, ChatGPT asserted that, as a principle, written consent was recommended. It emphasized the establishment of a process for voluntary participation based on ethical principles. ChatGPT stressed that the content of informed consent should not only outline the risks and benefits of the study but also include the disclosure of conflicts of interest. This response indicates that ChatGPT likely had an accurate understanding of ethical review-related laws and regulations. However, the initial response (Q3-1) maintained a similar level of detail, lacking specific considerations for patients with dementia. Conversely, when directly inquired about special considerations for dementia patients, they suggested the necessity of explaining the research to legal representatives and family members for obtaining consent. These responses align with reported key factors in the clinical challenges of dementia [29]. The results were interactive, revealing more detailed answers with an increased number of questions. In essence, it was confirmed that ChatGPT can provide more accurate and comprehensive responses through detailed inquiries.

Collectively, these findings indicate that ChatGPT possesses an understanding of ethical principles and can provide fundamental ethical responses to a variety of questions. However, the impact of machine learning was evident, with some responses being extreme, overly focused on specific ethical considerations, or generally superficial. ChatGPT itself acknowledged the limitation, stating that it cannot comprehensively address ethical considerations but can offer insights following ethical principles. Additionally, the phenomenon of repeated answers was observed, which was prominently confirmed in the results of this study. This suggests that machine learning-driven streamlining of responses may lead to specialization or extreme viewpoints on ethical matters. Moreover, this study’s results depend on the specific version of the large-scale language AI model used. Therefore, if the language model’s training data or algorithm is modified in the future, responses could vary, posing challenges to reproducibility. Additionally, while providing accurate responses is crucial in research ethics, large-scale language models can inherit biases, necessitating caution to prevent AI from perpetuating existing biases or introducing new ones. It is essential to consider generative AI while understanding these concerns. On the other hand, in recent years, AI has been actively used to address medical ethics issues through natural language processing [30] and to extract genetic medical problems using large-scale language models [31], highlighting the importance of ethical consultation [32, 33]. Based on these existing papers and the results of this study, there is a strong need to explore research ethics consultation using generative AI, and we must consider its future applications carefully. However, the ongoing debate on AI’s role in decision-making emphasizes the ultimate need for human experts [34]. Moreover, AI-driven research and development demand thorough ethical considerations. In response to Q5, ChatGPT clarified its role, stating “We can propose in line with ethical principles, but we cannot make judgments as we are AI.” This underscores that ChatGPT is an AI tool, highlighting the necessity for human decision-making and judgment. Based on these outcomes, it is apparent that existing generative AI can address basic ethical considerations in research; however, caution should be exercised in interpreting such responses. In the future, we believe that AI could serve as a valuable tool for ensuring subject protection by accurately identifying ethical issues. However, this study has limitations as the ethical validity of ChatGPT was not thoroughly examined using an evaluation tool, making the current results only indicative of ChatGPT’s responses. Additionally, the questions in this study were posed in a hypothetical research situation rather than an actual research plan. Consequently, with questions limited to 50 words or about 3–4 lines of text, it remains uncertain whether ethical considerations can be extrapolated from a comprehensive text such as a genuine research plan. The current study also lacked a robust framework to assess the ethical depth and relevance of the AI-generated responses. For example, conducting a comparative analysis between responses from the Human Ethics Committee and those generated by AI and exploring how AI-derived insights are integrated with human judgment, are areas for future study. Therefore, as demonstrated in this study, relying on AI for ethical considerations in medical research may have operational, legal, and ethical implications and must be approached with caution. Nevertheless, in exploring specific ethical aspects of research, there is potential to interactively confirm ethical points using generative AI such as ChatGPT. In recent years, reports have emerged on using AI trained in ethical principles for clinical ethics decision-making. While AI can provide efficient decision-making advice, it is sometimes inferior to human judgment [35]. Therefore, careful consideration will be necessary for future applications. However, we aim to explore further uses of AI to protect human subjects by considering the learning capabilities of generative AI and rigorously verifying its performance.

## Conclusion

In this study, we employed ChatGPT, a generative AI, to assess ethical issues within a specific research context. The results yielded basic responses generally aligned with international ethical guidelines; however, machine learning optimization revealed certain biased opinions. Although the use of generative AI for enumerating ethical issues is a novel approach, it suggests potential applications in protecting human subjects in the future. Future research should focus on evaluating the ethical validity of generative AI and exploring effective ways to integrate AI into the realm of research ethics.
