# Supplementary material for: Exploring ethical considerations in medical research: Harnessing pre-generated transformers for AI-powered ethics discussions
Source: PLoS One. 2025 Feb 3;20(2):e0311148. doi: 10.1371/journal.pone.0311148 (PMC11790142; doi:10.1371/journal.pone.0311148)
Supplement: Attachment — Submitted filename: Response to Reviewers.docx [file pone.0311148.s001.docx]

【Response to Reviewers】

Dear Dr. Emily Chenette,

Thank you for your ongoing consideration of our manuscript for publication in PLOS ONE. We appreciate the time spent by you and the reviewers and believe the revised manuscript is improved.

Below, we have addressed your and the reviewers’ comments.

--

Journal Requirements:

**1. Please ensure that your manuscript meets PLOS ONE's style requirements, including those for file naming.**

→We have responded.

**2. Thank you for stating the following in the Acknowledgments Section of your manuscript:**

**"This work was supported by SPIRITS（Supporting Program for InteRaction-based Initiative Team Studies）2022 of Kyoto University"**

**Please include your amended statements within your cover letter; we will change the online submission form on your behalf.**

→We have responded.

I also added the following to my cover letter:

Kyoto University Institute for the Future of Human Society Collaborative Research Project 2023.

**3.Please include this amended Role of Funder statement in your cover letter; we will change the online submission form on your behalf.**

→We have responded.

**4. We note that you have stated that you will provide repository information for your data at acceptance. Should your manuscript be accepted for publication, we will hold it until you provide the relevant accession numbers or DOIs necessary to access your data. If you wish to make changes to your Data Availability statement, please describe these changes in your cover letter and we will update your Data Availability statement to reflect the information you provide.**

→I have confirmed.

**5. Please include a copy of Table 1-5 which you refer to in your text on pages 10-11.**

→We have responded.

--

| Reviewer Comments | Response |
| --- | --- |
| Reviewer １ | |
| 1. Page 2 Line 39: The authors referred to Belmont Report and the Declaration of Helsinki as “universal indicators” which I think is not accurate. These are more of international regulations than indicators. This should be revised. | Thank you for pointing this out.  I revised it as follows.  universal indicators →　international regulations |
| 2. The authors use the term "Joint research" which I suggest should be revised to "collaborative research". | Thank you for pointing this out.  I revised it as follows.  "Joint research" →"collaborative research" |
| 3. Page 4 lines70-72: This is a factual statement that should be referenced. | I corrected the article and referenced it. |
| 4. Line 74-75: "There are many reports on the ethics of the use of AI in research [11] [12], | I decided it was unnecessary and deleted it. |
| 5. 75 but there is no report on how AI handles research ethics." What do the authors mean by "how AI handles research ethics?" That phrase does not seem to make a lot of sense. | I decided it was unnecessary and deleted it. |
| 6. The objective of the study is not concise and is poorly stated, as such, this makes it difficult to appreciate the authors arguments and line of thought. The objective should be recast. | Thank you for pointing this out.  I have revised the wording so that the purpose of this question is limited to "Thus, this study provided input summaries of various medical research into ChatGPT and examined whether interactive AI can discuss the ethical issues of research." |
| 7. The authors have outlined the questions they asked ChatGPT but they have not described how the data was analyzed. This aspect should be added to the methods section. | All the answers to the question were written down, a table was created, and the contents were examined by two researchers. I have added this information to the Methods. |
| 8. Line 228-231. Who was concerned? The authors or ChatGPT. | Shows the ChatGPT answer. In order to distinguish between the author and ChatGPT, the entire sentence has been corrected to use proper nouns. |
| 9. Overall, the discussion is written in poor English and difficult to follow. It is difficult to discern what message the authors would like to communicate to the reader. The discussion section is majorly a recap of the results with minimal actual discussion and interpretation of the results. | As much as possible, we have revised the discussion to make it easy to understand and reasonable. |
| 10. The description of the ChatGPT output in the discussion is poorly done and confusing. In some instances, it is difficult to differentiate between the authors opinions and the study findings. Further, there is inappropriate use of "it'", "we" and "they". | The output contents of ChatGPT have been clearly expressed in the text.  Specifically, I always used the subject of "ChatGPT" when answering ChatGPT. |
| 11. Line 254-257: "Taken together, these findings revealed that ChatGPT understood the ethical principles and can provide the most efficient answers to various questions. On the other hand, there were cases where the understanding was extreme, resulting in answers that focused on one part of ethical considerations or answers that were shallow overall". What is meant by efficient answers? What is meant by extreme understanding? There are two conflicting messages in the above statement. I suggest that more elaboration is given. The discussion should be improved. | English mistake. I fixed it as follows:  Taken together, these findings reveal that ChatGPT understands ethical principles and can provide the most basic ethical answers to various questions. In contrary, owing to the influence of machine learning, in some cases, responses were extreme, focused on some of the ethical considerations, or were generally shallow. |
| 12. The authors stated that "In this study, an existing interactive AI was used to examine possible issues related to subject protection in research". But this is different from the objective stated in the introduction. Authors also conclude stated that "We discovered the usefulness and challenges of using AI to assist in ethical review". However, these are not reported in the result section. Furthermore, the discussion is shallow on these aspects. The objective of the study should be consistent and in line with the conclusion | Thank you very much for your very important opinion. In the current situation, it was an expression that invites misunderstanding as a leap of logic. Changed the purpose at the beginning to whether or not to point out research ethics in generated AI. This time, I will state the results obtained with ChatGPT in the conclusion.  In other words, both the purpose and the conclusion have been revised. |
| Reviewer ２ | |
| Reviewer #2: Unfortunately I recommend rejection for this manuscript even though I think it was a great idea for a study which could potentially be very interesting. But as currently described and constructed it wasn't particularly well implemented. What I think it has shown is that the AI can identify key principles from some notable policies, and also potential risks/side effects from certain clinical procedures, but I am not sure what this has told us about the process (or potential of AI) of/in ethics review.  Although the intention was to initially establish the foundations of an ethics review by referring to the declaration of Helsinki and Belmont report in particular, the study 1) did not spend enough time/detail defining the content of a "good" research ethics review for subsequent comparison with the AI ethics review. Linked to this, 2) research ethics committees review voluminous, complex, information about a project, whereas this study only presented extremely short vignettes to the AI. Unfortunately this makes the current work of little relevance to "real" ethics reviews where the difficulty almost always lies in the contextual details of projects under review. As this study did not address this point in particular, I think it has missed the key thing that AI might be able to add - the ability to rapidly review large and complex study protocols and identify the key issues. Rather than provide short vignettes, the researchers really needed to give ChatGPT some extensive study documentation and then see whether it could analyse it rapidly and accurately, identifying relevant ethical issues.  Furthermore, this manuscript needs 3) substantial proof reading and editing. For instance, the introduction needs a major revision to more accurately reflect the current situation within research ethics reviews. There are also inaccuracies such as the World Health Organisation definitely does not "assure the quality of ethics reviews", and while the examples of accreditation are interesting, there is a substantial literature about ethics committee consistency that has not been referenced. Likewise there are many guidance documents that list the questions that ethics committees need to ask when reviewing projects - it would be nice to have seen at least some of these referenced and perhaps the questions listed in a table/figure (going some ways to address my concern number 1, stated above).  Finally in a number of places there are statements about ChatGPT "understanding" ethical principles. I would question this language. Maybe it identified principles, but understanding is a very different (and difficult to define) issue. | Thank you for your valuable opinion.  As you said, I understand that there is insufficient verification to compare with the Ethics Committee.  In this rivision paper, referring to this opinion and the opinions of other reviewers, we will focus on "Can we extract ethical opinions in research using generative AI?"  Significant revisions have been made to the portion of the current description showing a comparison with the actual Ethics Committee.  Added the question of whether AI understands ethical principles, which was a concern in the comments, to discussion of the study. |
| Reviewer ３ | |
| Major: the major issue of the article has to do with the fact that the methodology used to judge the quality of ChatGPT's responses is not identified in the methodology section nor made explicit in the succeeding sections. As it is, the reader is left guessing whether ChatGPT's responses were pitted against those of the researchers. If indeed so, how sufficient is this methodology for evaluation considering that the point of comparison should be research ethics committee members and their review processes? | Thank you for your valuable feedback. Since it is not a level of verification that can be compared with direct ethical review, we have organized the purpose and conclusion and made significant revisions. |
| 1. line 43-45: "The assurance of the quality of ethics committee reviews has long been carried out by the World Health Organization, an international human subject protection program." -- WHO is not an international human subject protection program, though this might be a grammatical issue. | It was a grammatical error, so I fixed it as follows.  In an effort to ensure the quality of Ethics Committee reviews, the World Health Organization has issued guidelines on review quality and practices. |
| 2. line 54-54: "it is considered most important for the members of each committee 'to provide many opinions for subject protection'" -- The idea here is contestable. It is not the number of opinions ("provide many opinions) but whether the opinions are exhaustive to have captured all the relevant ethical concerns. | As you said, I have modified it as follows.  On the other hand, to solve the quality problem of actual ethics committees, it is considered most important for the members of each committee to “"Provide a comprehensive opinion of ethical considerations for the protection of human subjects.".” |
| 3. line 63-65: "However, in humanities and social sciences, because there are many ethics committees, the quality of  65 each committee is inconsistent, and the medical field is no exception" -- Can we realistically say that the number of ethics committees correlate to the quality of review? | Sorry for the misunderstanding caused by my English mistake. We have corrected it as follows.  However, owing to the current situation in Japan, there are suspicions regarding the declining quality of ethics review in the humanities and social sciences owing to the disparity in opinions and review resources because of the excessive establishment of ethics committees. |
| 4. line 65-67: Moreover, in joint research, the general rule is to conduct a collective review by asking the opinions of  one representative ethics committee,..." -- Please provide the context. Note that this is not a generalizable procedure. | I have inserted a paper and improved the description to show that the number of countries that have adopted collective review is increasing. |
| 5. line 69-70: "Therefore, it is important to have a common tool for each committee and a mechanism that allows a certain amount of opinions to be expressed" -- That a "common tool" is necessary is an assumption that needs some form of justification. Why are common standards not sufficient? | As you pointed out, there are various possible cases, so we have revised the description. |
| 6. line 70-72: "Opinions expressed uniformly and homogeneously by natural language processing artificial intelligence (AI) are believed to contribute to improving the quality of ethics committees." -- Provide reference. | This is the opinion of the author, so I have revised it as follows.  Furthermore, AI and machine learning can reportedly prevent human errors in medical diagnosis [10]. An AI that detects such errors could be useful, for example, in enumerating ethical points from a research protocol. |
| 7. line 72-74: Furthermore, AI and machine learning were reported to prevent human errors in medical diagnosis and ethical review" [10] -- The reference cited did not include "ethical review", contrary to what the authors claim. | As you pointed out, [10] does not include ethical review, so I deleted the "ethical review" part.  I have added the following sentences.  --  An AI that detects such errors could be useful, for example, in enumerating ethical points from a research protocol. |
| 8. line 162-166: It seems that the researchers asked a question which was interpreted differently by ChatGPT. The researchers were probably asking about ChatGPT's knowledge of the "Japanese Ethical Guidelines for Medical and Health Research Involving Human Subjects," while ChatGPT understood the question to mean, "what are the ethical guidelines for medical and health research involving human subjects", i.e., a more generic question instead of the more specific question relating to ChatGPT's familiarity with the Japanese guideline. This raises the claims related to this answer as invalid. | We also asked whether or not they had studied, and received an answer that they had. I've added it to the Method section and Discussion to make that clear. Also, I added the following sentences so that it would not be an excessive expression.  --  However, it presently remains unclear whether it is possible to apply ethical principles to various research contents and respond flexibly about the topic of participant protection only with this understanding. |
| 9. In the Discussion section, was the word "we" used to refer to ChatGPT's responses? | I clarified the distinction between ChatGPT and the author.  Specifically, when referring to ChatGPT, I expressed it with a proper noun. |
