# Supplementary material for: Exploring ethical considerations in medical research: Harnessing pre-generated transformers for AI-powered ethics discussions
Source: PLoS One. 2025 Feb 3;20(2):e0311148. doi: 10.1371/journal.pone.0311148 (PMC11790142; doi:10.1371/journal.pone.0311148)
Supplement: Attachment — Submitted filename: Response to Reviewers.docx [file pone.0311148.s002.docx]

【Response to Reviewers】

Dear Dr. Emily Chenette,

Thank you for your ongoing consideration of our manuscript for publication in PLOS ONE. We appreciate the time spent by you and the reviewers and believe the revised manuscript is improved. We have made extensive and sincere revisions to the manuscript.

Below we discuss responses to reviewer comments.

| Reviewer Comments | Response |
| --- | --- |
| Reviewer＃１ | |
| 1. I have a problem with the title. "Ethics of medical research" is a bit vague. Do the authors mean ethical issue in medical research? Ethical considerations in medical research? | Thank you for pointing this out.  I revised it as follows.  "Ethics of medical research"→”ethical considerations in medical research” |
| 2. The abstract is not informative at all. For instance, the objective is poorly stated, the methods are missing, the primary results are not clear, and the conclusion is more of a recommendation. I suggest that the authors structure the abstract in the conventional way i.e., Introduction, Methods, Results and Conclusion. | Thank you for your important comments. As suggested, we have reorganized the article into an introduction, methods, results, and conclusion. In addition, as a result of reconsideration, we have made significant changes to the entire text. |
| 3. Almost all reviewers felt that the introduction needed major revisions however the authors did not do so. The introduction does not flow well. In line 40, the authors mention the Belmont report and Declaration of Helsinki. In the next sentence they mention laws and regulations; however, these are not legal documents. They are ethical guidelines. | Thank you for your important feedback. An error occurred during English proofreading, and the words "laws and regulations" were used multiple times. We have reviewed the relevant sections and revised the ethical guidelines. |
| 4. Line 46-47 "World Health Organization has issued guidelines on review quality and practices"; references for these guidelines should be cited. | Since the description was insufficient, we have provided a detailed description and cited the guidelines as a reference. |
| 5. All abbreviations should be written in full the first time they are used in the main body of the manuscript. | We reviewed the manuscript and ensured that if we used an abbreviation, we wrote the full word first. |
| 6. Line 51-52 "In Japan, the Ministry of Health, Labor and Welfare implemented an ethics committee certification system". But no elaboration is given, on reference is cited etc. The introduction is full of these isolated statements with no logical flow, as such, making it difficult to follow. The authors should avoid these isolated statements. More information should be provided on this system since this is a Japanese study. | Added information and reference URL for this project as a citation. I created a connection with the previous sentence to avoid becoming an orphan sentence. |
| 7. Line 56-57: “Provide a comprehensive opinion of ethical considerations for the protection of human subjects.” Where was this quote obtained? The authors should provide a reference and page number. | Added reference papers. For a long time, the opinions of committee members have been considered important, and for example, in the field of genetics, there are many opinions regarding considerations regarding genetics, but there are no opinions regarding pain associated with testing, etc., which has been raised as a problem in papers. Furthermore, in the study of single IRBs, there are doubts that the quality of the committee members influences the review, and the opinions of the committee members are treated as an important factor in the quality of the review. I've added those papers to my bibliography. |
| 8. 8. Line 61-62: "In addition, in collaborative research involving multiple institutions, a single ethics committee is the mainstream for collective review". I do not understand this statement. Do the authors refer to a research ethics committee of record? Centralized ethics review boards for multi-centre studies? The reference cited is more than 10 years old. Do the recommendations of in that referenced study still hold? | Research funded by American NIH grants and research that complies with Japan's ethical guidelines are subject to single IRB review. In Japan, from 2022 onwards, all examinations will be single IRB review. To avoid misunderstandings, I have referred to some countries, including Japan, as it appears to be shared worldwide. Also, as you pointed out, the Single IRB has been controversial and is still being debated to ensure a more friendly Single IRB review. I have also added the advantages and disadvantages along with the cited references. |
| 9. 9. Line 64 has a problem; it should be revised. | Fixed the duplicate parts. I am terribly sorry. |
| 10. It seems reference 9 is in Japanese, this should be stated in the reference list. | Thank you for your confirmation. Added to reference list. |
| 11. Line 76-78: "Therefore, we aimed to investigate whether it is possible to extract ethical points in various research fields using existing interactive AI". I am not sure what "ethical points" the authors are referring to in this objective. The objective is not SMART and should thus be rephrased. | In the introduction, we have improved the flow of sentences up to stating the purpose. Specifically, "Deterioration in the quality of IRB reviews" → "Past efforts to improve quality" → "Need to diversify committee members' opinions" → "Past advances in generative AI" → We have organized the overall flow of "Can AI point out the ethical considerations in medical research?" Based on the above, I have briefly stated the purpose in the last line. |
| 12. Line 90-91: "Thus, this study provided input summaries of various medical research into ChatGPT and examined whether interactive AI can discuss the ethical issues of research". Which of the two statements was the objective of the study? | I deleted it because it was ambiguous. As per answer 11, the purpose section has been extensively updated. |
| 13. Line 105-108: "In addition, as this study used AI to obtain answers of virtual questions, which were created with research content including ethical concerns considered by the author, and does not involve human participants, the need for approval from an ethics committee was waived". Does the above statement allude to ethical considerations for this study? If so, it should be revise by first stating that the study was exempted from further REC review because of A, B, C etc. The REC reference number for the waiver should be quoted. | It is also possible that the misunderstanding occurred due to an issue with English proofreading. As you pointed out, we have amended the article to include the reasons for exemption from ethics review. We have confirmed that there is no exemption number for cases that are not covered by domestic ethical guidelines and do not require ethical review, as our university's Ethics Committee does not issue a certificate of exemption from ethical review. |
| 14. Line 125-128: “Q2-1 A study of 50 dementia patients, we will analyze specific proteins in the blood of cognitive function tests and blood samples, and conduct research to confirm the correlation between cognitive function and specific proteins”. What are the ethical considerations required in this case. I think some of the questions that ChatGPT was asked did not make a lot of sense, unless there was a problem translating them to English. Do cognitive function tests have blood like it is stated in the above statement? | Thank you for your important feedback. In this case, I adopted this question because if I use a blood sample, I want to consider whether it is a blood sample or an existing sample. Although the wording is ambiguous, we would like to use the answers to such questions as part of the results, so we will use this question. |
| 15. ChatGPT was asked these questions, but nothing is mentioned about how the responses were analysed. Reviewers requested for improvements in the methodology, but this was not satisfactorily done. The methods are still poorly described. | Thank you for your frequent comments. We have received specific opinions from other reviewers, so we have updated the content. In addition, matters that could not be elucidated using the methods of this study were also noted in the discussion section as limitations of this study. |
| Results  16. From the way results are presented, in some instances, It is difficult to tell whether the results are from ChatGPT or are opinions of the authors. Result presentation should be improved. | Thank you for pointing this out.  I changed the description to make it clear that this is ChatGPT's answer. |
| 17. Line 295: "Therefore, it was suggested that the opinion of ethical considerations, for which answers were streamlined by machine learning, lead to extreme answers." What are extreme answers? | An "extreme answer" indicates that it is not a comprehensive opinion, but is limited to a few opinions. I added words to make it clearer. |
| 18. The conclusion feels blunt and noncommittal. What are the implications of the study findings to practice, policy, and research? | Thank you for pointing this out. Added a description of how the research results will affect practice, policy, and research. We have revised it significantly. |
| 19. What are the strengths and limitations of the study? | Added text to the manuscript regarding the limitations of this study. The breaking point is that ChatGPT's system evaluation is not rigorous, so it should be treated as just a ChatGPT answer. Furthermore, since the number of sentences in this question is small, it is unclear whether it can be applied to an actual research plan level. The advantage is that this is an unprecedented effort, and generative AI could be a useful tool for considering partial ethical issues. |
| Reviewer #4 | |
| Abstract:  • I believe that the abstract can be further improved. Ensure the abstract concisely summarizes the key points of the study, including objectives, methods, results, and conclusions. | Thank you for your important comments. As suggested, we have reorganized the article into an introduction, methods, results, and conclusion. In addition, as a result of reconsideration, we have made significant changes to the entire text. |
| Introduction:  • The introduction does a good job establishing the rationale for exploring AI in ethics reviews. To take it a step further, provide more specifics on the documented quality issues with committee reviews (e.g. statistics on decreasing thoroughness, common oversights, etc). This will further strengthen the justification.  • When introducing ChatGPT, give 1-2 sentences on the basics of how it was developed/trained and why it represents a more advanced AI system. This context will help readers new to ChatGPT.  • Expand on the background more to provide a more comprehensive context for the study. | In the introduction, we have improved the flow of sentences up to stating the purpose. Specifically, "Deterioration in the quality of IRB reviews" → "Past efforts to improve quality" → "Need to diversify committee members' opinions" → "Past advances in generative AI" → We have organized the overall flow of "Can AI point out the ethical considerations in medical research?"  We have prepared a description of how to use ChatGPT that we believe can be applied to medical research by expanding the background and improving the description. Regarding issues related to the quality of the committee's review (oversight, lack of thoroughness), there is little objectively disclosed data, so we have organized the text to make it clear that the opinions of the committee members are more important. |
| Methods:  • Clarify the rationale behind choosing the specific version of ChatGPT used in the study.  • Expand on the data analysis process. Specifically touch on:   How were ChatGPT's responses evaluated for accuracy, depth, and ethicality? What metrics were used?   Did the two experts review the responses independently first and then come together?   Was there a standardized rating scale or an open-ended discussion?   How were differences in opinion between the experts reconciled? | Added reason for version selection. Added description regarding data accuracy evaluation and analysis. It added that an open-ended discussion was held to conduct exploratory research.  Additionally, although discussions were held, it was not possible to examine the validity of ChatGPT in detail, which was added as a limitation of this study. |
| Discussion:  • Enhance the discussion of implications, limitations, and potential applications of AI in ethics reviews.  • Address the variability in ChatGPT's responses and discuss how this impacts its potential utility in ethical reviews. | We have added the limitations of this study to the discussion. Furthermore, given its limitations, we enumerated the potential usefulness of generative AI in reconsidering ethical issues in research. |
| Conclusion:  • Include recommendations for future research and potential improvements in AI for ethical reviews. | The conclusion section has been significantly revised to incorporate your comments. |
| Furthermore:  • Throughout the manuscript, attention to grammatical precision and clarity is needed.  • The manuscript would benefit from thorough proofreading to correct minor language errors and enhance readability. | Thank you for your feedback.  Both the first edition and the revision were proofread by an English proofreading company, but we apologize for not being able to provide a sufficient level of English. Once again, we outsourced the work to a proofreading company and requested a thorough English proofreading. |
