# Supplementary material for: Exploring ethical considerations in medical research: Harnessing pre-generated transformers for AI-powered ethics discussions
Source: PLoS One. 2025 Feb 3;20(2):e0311148. doi: 10.1371/journal.pone.0311148 (PMC11790142; doi:10.1371/journal.pone.0311148)
Supplement: Attachment — Submitted filename: Response to Reviewers.docx [file pone.0311148.s003.docx]

【Response to Reviewers】

Dear Dr. Emily Chenette,

Thank you for your ongoing consideration of our manuscript for publication in PLOS ONE. We appreciate the time spent by you and the reviewers and believe the revised manuscript is improved. We have made extensive and sincere revisions to the manuscript.

Below we discuss responses to reviewer comments.

| Reviewer Comments | Response |
| --- | --- |
| Reviewer＃5 | |
| I was invited to review the manuscript after the second round of reviews and will try to be as concise as possible.  The topic is very up-to-date and there are some new papers on ChatGPT and bioethics. The article could be placed somewhat more in context of the discussion by adding additional references. The issue of The American Journal of Bioethics, Volume 23, 2023 - Issue 10 could be helpful, for instance. If these papers that were published (I guess) after submitting the manuscript have too much of an effect on the central argument they can still be noted as issues in need for further research.  While not referring to ChatGPT, there is an interesting paper that relates to this topic:  Lukas J. Meier, Alice Hein, Klaus Diepold & Alena Buyx (2022) Algorithms for Ethical Decision-Making in the Clinic: A Proof of Concept, The American Journal of Bioethics, 22:7, 4-20, DOI: 10.1080/15265161.2022.2040647  Here the authors drop the “justice” element, as it was “too difficult” to include. It appears that AI models are better at applying some principles (especially informed consent) than others. This fits well with the discussion by the authors.  Furthermore, there is a discussion in applied ethics stating that ethics should be more than a mere tick box exercise. While it is useful to have tick boxes to avoid missing standard issues, they do not invite to weight different factors. Much of the discussion refers to this problem and it would be important to put the discussion in light of this older debate on doing applied ethics (or methods in applied ethics). | Thank you very much for your review.  I learned a lot from the advice I received regarding the discussion.  I read the paper you presented and added it to the discussion section regarding the advantages and disadvantages of using AI for ethical decision making.  Other reviewers also raised concerns about the use of AI, which were limitations of this study, and added them to the discussion.  thank you for understanding. |
| Reviewer #6 | |
| Reviewer #6: Thank you for the opportunity to review this article!  The authors present an interesting and timely research idea. No doubt that the potential use of AI in research ethics review process will be (and already is) intensively discussed.  Nevertheless, the research study and the paper include significant shortcomings as already pointed out by previous reviewers. First and foremost, the methodology of the study is rather vague as has been pointed out by Reviewer 1. The data in the appendix show that the authors have used very short fictional summaries of research studies (one sentence) to inquire ChatGPT about ethical issues in these scenarios/questions. It is not clear whether these scenarios/questions were originally written in Japanese or English and what was the language of conversation with ChatGPT. In any case, this process is not comparable to a real ethics review where REC members are reviewing the protocol, informed consent form and other documents in full. In my view, to make any conclusions about the capacity of AI to discuss ethical issues in research protocols, the study should be built on real documents or at least fictional research protocols and informed consent forms at full length. Additionally, the scenarios/questions as they are presented in the appendix include mistakes and ambiguities. It is also not justified why specifically ChatGPT was used for generating answers since there are many different AI tools available for generating texts. It is not clear how the results were analyzed. The only indication of the approach to analysis are the phrases “two research ethics experts confirmed and examined the contents” and “then engaged in open-ended discussions about the content”. To my knowledge, it is not a methodical approach to analysis. For strong and rigorous analysis much more detailed method of analysis has to be applied, and there are many approaches in qualitative analysis that might be successfully used.  I also concur with the previous reviewers that the introduction and discussion do not flow well and are rather fragmented. Unfortunately, the two attempts to improve the text following the reviewers' comments do not seem to be fully successful. I guess that one of the reasons for that is the shortcomings in the methodology mentioned above. Consequently, the conclusions are very vague and it is not clear how they are supported by the data.  My suggestion to the authors is to continue the research by developing more detailed research idea and aim, implementing a new research study and writing a new, much stronger paper. | Thank you very much for your review.  I learned a lot from the advice I received as a result of this review.   1) First and foremost, the methodology of the study is rather vague as has been pointed out by Reviewer 1. The data in the appendix show that the authors have used very short fictional summaries of research studies (one sentence) to inquire ChatGPT about ethical issues in these scenarios/questions. It is not clear whether these scenarios/questions were originally written in Japanese or English and what was the language of conversation with ChatGPT.   →  Thank you for pointing out the methods section.  The methods section has been significantly revised.  First, the short sentences in question were written in English, taking into consideration the difficulty of processing long sentences in generative AI models and the fact that learning conditions differ depending on the language.  As you pointed out, what actually took place in this study was to discuss research ethics in a short dialogue format, rather than a review at the research plan level, so we reorganized the method text.   2) In any case, this process is not comparable to a real ethics review where REC members are reviewing the protocol, informed consent form and other documents in full. In my view, to make any conclusions about the capacity of AI to discuss ethical issues in research protocols, the study should be built on real documents or at least fictional research protocols and informed consent forms at full length.   →  This research was merely an exploratory study looking at the answers of existing generative AI, and due to the performance of the generative AI, it was not possible to process actual research plans or explanatory documents.  Therefore, as you pointed out, it is far from the REC process.  I have added this point as a limitation of the research.   3) It is also not justified why specifically ChatGPT was used for generating answers since there are many different AI tools available for generating texts.   →  Thank you for pointing this out.  We are aware that there are currently countless generative AIs around the world that implement LLM, but this paper was first submit in April 2023, and we have been conducting research since shortly after ChatGPT was released. Ta. In addition, the reason for choosing ChatGPT is that it is a generative AI that is used by many users, as well as having been published in academic papers and undergoing numerous verifications. I have included these as reasons for my selection.   4) It is not clear how the results were analyzed. The only indication of the approach to analysis are the phrases “two research ethics experts confirmed and examined the contents” and “then engaged in open-ended discussions about the content”. To my knowledge, it is not a methodical approach to analysis. For strong and rigorous analysis much more detailed method of analysis has to be applied, and there are many approaches in qualitative analysis that might be successfully used.   →  Thank you for pointing this out.  He added that the analysis method also considered consistency with existing papers.  Also, as you pointed out, this survey does not allow for qualitative analysis. The reason is that this is a challenging exploratory study, and we believe that only the answers of the current generation of AI need to be discussed immediately. However, the lack of qualitative analysis is an issue for this study, so we added it as a main research issue in the discussion section. |
| Reviewer #7 | |
| Reviewer #7: Thank you for allowing me to review this manuscript. The topic is interesting and timely. Kindly find my few comments below. • I think the title should be modified. The second part of the title is not complete “Utilizing a generative pretrained transformer” for what? It can be, for example “Utilizing a generative pretrained transformer to address this issue.” • The same applies for the short title. • Abstract o The goal of the study was to explore the use of ChatGPT “to enumerate ethical issues in medical research.”. I am not sure if the word “enumerate” is the correct choice. Enumeration is related to counting or listing items, and I don’t think this is the case here. Maybe “discuss” or other word will be more suitable. Please check. • Introduction o The previous point applies also for the introduction, page 4, line 80. o Page 4, line 83, the authors mentioned that “Developed by Open AI, ChatGPT is capable of challenging incorrect assumptions.” I think this statement should be modified, because the chatbot still has limitations regarding accuracy. | Thank you very much for your review.  I learned a lot from the advice I received as a result of this review.  • I think the title should be modified. The second part of the title is not complete “Utilizing a generative pretrained transformer” for what? It can be, for example “Utilizing a generative pretrained transformer to address this issue.”  • The same applies for the short title.  →  Thank you for your feedback.  We have corrected the title and short title according to your suggestions, so please check them.  • Abstract  o The goal of the study was to explore the use of ChatGPT “to enumerate ethical issues in medical research.”. I am not sure if the word “enumerate” is the correct choice. Enumeration is related to counting or listing items, and I don’t think this is the case here. Maybe “discuss” or other word will be more suitable. Please check.  →  As you pointed out, "discuss" was appropriate, so we changed it to "discuss".  • Introduction  o The previous point applies also for the introduction, page 4, line 80.  →  As you pointed out, "discuss" was appropriate, so we changed it to "discuss".  o Page 4, line 83, the authors mentioned that “Developed by Open AI, ChatGPT is capable of challenging incorrect assumptions.” I think this statement should be modified, because the chatbot still has limitations regarding accuracy.  →  Thank you for your important comments.  As you said, we have added to the text that there is still room for discussion regarding the system for generating AI responses. |
| Reviewer #8 | |
| Reviewer #8: Abstract:  The manuscript presents an exploratory study on the use of generative AI, specifically ChatGPT, to enumerate ethical considerations in medical research. The authors aimed to assess the AI's ability to identify ethical issues in various research contexts, using the AI's responses to structured questions that simulate real-world research scenarios. While the innovative approach and attempt to address the challenges of ethical review in medical research is commendable, several major concerns and limitations warrant attention.  Major Concerns:  -The study shows that while ChatGPT can enumerate basic ethical considerations, it struggles with the depth and nuanced understanding required for comprehensive ethical analysis. This limitation is particularly troubling in complex cases or where subtlety in ethical reasoning is critical. AI's inability to provide specific measures for psychological considerations or broader ethical issues underscores this shortcoming.  -Also, why was GPT3.5 and now GPT4 chosen? IMO, this does not make much sense.  Bias and machine learning optimization:  -The manuscript acknowledges the potential for biased opinions due to machine learning optimization, but does not thoroughly explore or address how these biases might impact the ethical review process. Given the critical importance of unbiased ethical considerations in medical research, the potential for AI to perpetuate existing biases or introduce new ones is a significant concern.  Methodological limitations:  -The methodology relies heavily on the current capabilities of AI without a robust framework for evaluating the ethical -depth or relevance of its responses. The lack of comparative analysis with human ethics committee decisions or discussion of how AI-derived insights integrate with human judgment is a notable gap.  -A more critical examination of areas where ChatGPT falls short, especially in complex ethical scenarios, would provide a more balanced view of its applicability in ethical reviews.  -The discussion reiterates results without fully leveraging them to explore the broader implications for AI in ethical decision-making.  Practical implications and integration challenges:  -While the manuscript suggests potential applications of generative AI to support ethical review, it does not adequately address the practical challenges of integrating AI insights into existing ethical review frameworks. The operational, legal, and ethical implications of relying on AI for ethical considerations in medical research are not explored in depth.  Reproducibility and transparency:  -The reproducibility of the study is questionable given the evolving nature of AI models such as ChatGPT. Changes to the model's training data or algorithms could significantly alter its responses over time, affecting the study's relevance and applicability in future research contexts.  Minor:  -Discussion of variability in ethical guidelines: There is insufficient discussion of the variability of ethical guidelines across countries, and how this variability affects the AI's ability to provide universally applicable ethical insights.  Overall assessment:  While the study presents an innovative approach, the implications of the findings for practical application in the ethical review process are not convincingly demonstrated. Also, the manuscript seems to be written by GPT in large parts, indicated by GPT-typical phrases, e.g. "realm of".. Overall, I am not convinced of the scientific benefit of this work. | Thank you very much for your review.  I learned a lot from the many comments that get to the heart of the matter.  As you have pointed out, this paper shows that, in a sense, it is difficult to discuss research ethics with current generative AI.  Please note that we do not promote the use of ChatGPT, but rather that we aim to quickly introduce the current level of generative AI to the world and hope for further development in the future.  -Also, why was GPT3.5 and now GPT4 chosen? IMO, this does not make much sense.  →  Thank you for pointing this out.  We are aware that there are currently countless generative AIs around the world that implement LLM, but this paper was first submit in April 2023, and we have been conducting research since shortly after ChatGPT was released. Ta. In addition, the reason for choosing ChatGPT is that it is a generative AI that is used by many users, as well as having been published in academic papers and undergoing numerous verifications. We also chose version 3.5 because it is widely available for free and has the largest number of end users among generative AIs. We have added the reasons for these choices to the methods section.  -The manuscript acknowledges the potential for biased opinions due to machine learning optimization, but does not thoroughly explore or address how these biases might impact the ethical review process. Given the critical importance of unbiased ethical considerations in medical research, the potential for AI to perpetuate existing biases or introduce new ones is a significant concern.  →  Thank you for your important comments.  In this case, as you pointed out, biases are also learned, so we have added a new note in the discussion section to note that there is a concern that the AI could state existing biases or create new ones. .  -The methodology relies heavily on the current capabilities of AI without a robust framework for evaluating the ethical -depth or relevance of its responses. The lack of comparative analysis with human ethics committee decisions or discussion of how AI-derived insights integrate with human judgment is a notable gap.  →  Thank you for your important comments.  In this case, there was a lack of a framework for considering the ethical depth and relevance of AI responses, so we have added this to the discussion section as a limitation of the research.  We have also added that it has not been possible to compare and consider human responses.  Since this is currently an exploratory study, we have decided to address this issue as a future issue.  -A more critical examination of areas where ChatGPT falls short, especially in complex ethical scenarios, would provide a more balanced view of its applicability in ethical reviews.  →  Thank you for your feedback.  In this revision, we have made significant additions to the effect that the use of LLM's generation AI needs to be proceeded with caution and that the current GPT answers need to be critically examined.  While the manuscript suggests potential applications of generative AI to support ethical review, it does not adequately address the practical challenges of integrating AI insights into existing ethical review frameworks. The operational, legal, and ethical implications of relying on AI for ethical considerations in medical research are not explored in depth.  →  Thank you for your important comments.  At this stage, we understand that actual operation is a long way off.  Also, as you point out, the operational, legal and ethical implications need to be considered.  We have added this to the discussion section as a limitation of this study.  -The reproducibility of the study is questionable given the evolving nature of AI models such as ChatGPT. Changes to the model's training data or algorithms could significantly alter its responses over time, affecting the study's relevance and applicability in future research contexts.  →  As you said, I added to the discussion section that the reproducibility of answers can change depending on the learning status of LMM, and that it is necessary to understand this and carefully consider the answers.  -Discussion of variability in ethical guidelines: There is insufficient discussion of the variability of ethical guidelines across countries, and how this variability affects the AI's ability to provide universally applicable ethical insights.  →  I have organized and added this as a future issue. |
