# Supplementary material for: Exploring ethical considerations in medical research: Harnessing pre-generated transformers for AI-powered ethics discussions
Source: PLoS One. 2025 Feb 3;20(2):e0311148. doi: 10.1371/journal.pone.0311148 (PMC11790142; doi:10.1371/journal.pone.0311148)
Supplement: Attachment — Submitted filename: Response to Reviewers.docx [file pone.0311148.s004.docx]

【Response to Reviewers】

Dear Dr. Emily Chenette,

Thank you for your ongoing consideration of our manuscript for publication in PLOS ONE. We appreciate the time spent by you and the reviewers and believe the revised manuscript is improved. We have made extensive and sincere revisions to the manuscript.

Below we discuss responses to reviewer comments.

| Reviewer Comments | Response |
| --- | --- |
| Reviewer＃1 | |
| Thanks for endeavoring to address the numerous comments from the reviewers. The utility of AI in research ethics review is questionable and more research is needed. | Thank you very much for your understanding. We greatly appreciate your feedback on the limitations of our study. We will consider these points carefully in future research, aiming to collect more detailed data and apply more rigorous methodologies to address these issues. |
| Reviewer #6 | |
| Reviewer #6: Thank you for the response to my comments!  As I noted in my previous review, the research idea presented by the authors is interesting and timely. Nevertheless, the methodology of the research study is vague, and the details added in this revision still do not allow a full understanding of the methods used for data analysis. For example, the sentence “Two research ethics experts then engaged in open-ended discussions about the content” cannot be viewed as a detailed description of the method of analysis.  Also, as I mentioned in the previous review, the scenarios/questions asked to ChatGPT are very short and are not comparable to a real ethics review. As a result, the ChatGPT answers are very short and uniform, for example, the paragraph about informed consent or vulnerability is almost identical for all scenarios. The authors have tried to analyse the ChatGPT responses as various individual responses for each case, however, it might be worth analysing the overlaps between the answers which might lead to the conclusion that the answers are constantly repeated.  Additionally, I still hold the view that the discussion and conclusions are vague. For example, the authors write in the discussion that “it remains unclear whether it can apply ethical principles to diverse research scenarios and respond flexibly to subject protection opinions”, meaning that additional research and most likely different methodology is needed to answer this question. The current version of the conclusion does not bring anything new to the scientific discussion. Why not do further research, generate more data, apply more rigorous methodology, publish more detailed results and meaningful conclusions? My suggestion to authors would be to develop this research idea and do additional research, especially taking into account the limitations of this study indicated by the authors. | Thank you very much for your thoughtful and detailed feedback on our manuscript.  As you noted in your previous review, we appreciate your recognition of the interest and timeliness of our research idea.  We understand and acknowledge your concerns regarding the vagueness of the research methodology and the insufficient details provided in our revision. We recognize that the sentence “Two research ethics experts then engaged in open-ended discussions about the content” does not provide a comprehensive description of the analysis method. We have addressed this by including discussions based on previously published studies to clarify the points discussed by the two researchers. In future revisions, we will aim to include a more detailed explanation of our methodology to enhance clarity and understanding.  Regarding the scenarios and questions posed to ChatGPT, we agree that they are relatively short and not fully comparable to a real ethics review. This limitation has resulted in ChatGPT's responses being very short and uniform, as you have observed. We acknowledge the value in analyzing the overlaps between these responses to better understand the phenomenon of repeated answers. In our discussion of the impact of machine learning, we have now explicitly noted that the phenomenon of repeated responses was prominently confirmed in the results of this study. This is an important point that we will consider in our future research to provide more robust and nuanced analyses.  We also appreciate your insights on the discussion and conclusions being vague. As you rightly pointed out, additional research and potentially different methodologies are necessary to determine whether ChatGPT can apply ethical principles to diverse research scenarios and respond flexibly to subject protection opinions.  However, a human being can consider a variety of ethical issues even when asked simple questions like those used in this study. Meanwhile, the interactive results using GPT-3.5 reveal many of the limitations of machine learning. We believe there is great value in documenting these in a scientific paper. We hope you understand this.  As I mentioned in the previous revision, this paper was submitted in April 2023, shortly after OpenAI released ChatGPT. Applying GPT-3.5 to research ethics is unprecedented, and we believe that this paper's appeal lies in explicitly examining various challenges and limitations in abstract questions like those in this study. We hope that this revised version and this response will be better understood.  Thank you again for your valuable feedback. We are committed to addressing these limitations in our ongoing and future research to develop more detailed and meaningful results and conclusions. |
