# Supplementary material for: Exploring ethical considerations in medical research: Harnessing pre-generated transformers for AI-powered ethics discussions
Source: PLoS One. 2025 Feb 3;20(2):e0311148. doi: 10.1371/journal.pone.0311148 (PMC11790142; doi:10.1371/journal.pone.0311148)
Supplement: Peer review history

# Peer review: Decision Letter 0

**DOI:** 10.1371/journal.pone.0311148.r001

**Authors:** Sally Mohammed Farghaly

1 Jun 2023

PONE-D-23-12275Can artificial intelligence point out the ethics of medical research?: Using a generative pretrained transformerPLOS ONE

Dear Dr. Mori,

Thank you for submitting your manuscript to PLOS ONE. After careful consideration, we feel that it has merit but does not fully meet PLOS ONE’s publication criteria as it currently stands. Therefore, we invite you to submit a revised version of the manuscript that addresses the points raised during the review process.

Please submit your revised manuscript by Jul 16 2023 11:59PM. If you will need more time than this to complete your revisions, please reply to this message or contact the journal office at plosone@plos.org. When you're ready to submit your revision, log on to https://www.editorialmanager.com/pone/ and select the 'Submissions Needing Revision' folder to locate your manuscript file.

Please include the following items when submitting your revised manuscript:

- A rebuttal letter that responds to each point raised by the academic editor and reviewer(s). You should upload this letter as a separate file labeled 'Response to Reviewers'.
- A marked-up copy of your manuscript that highlights changes made to the original version. You should upload this as a separate file labeled 'Revised Manuscript with Track Changes'.
- An unmarked version of your revised paper without tracked changes. You should upload this as a separate file labeled 'Manuscript'.

If you would like to make changes to your financial disclosure, please include your updated statement in your cover letter. Guidelines for resubmitting your figure files are available below the reviewer comments at the end of this letter.

If applicable, we recommend that you deposit your laboratory protocols in protocols.io to enhance the reproducibility of your results. Protocols.io assigns your protocol its own identifier (DOI) so that it can be cited independently in the future. For instructions see: https://journals.plos.org/plosone/s/submission-guidelines#loc-laboratory-protocols. Additionally, PLOS ONE offers an option for publishing peer-reviewed Lab Protocol articles, which describe protocols hosted on protocols.io. Read more information on sharing protocols at https://plos.org/protocols?utm_medium=editorial-email&utm_source=authorletters&utm_campaign=protocols.

We look forward to receiving your revised manuscript.

Kind regards,

Sally Mohammed Farghaly

Academic Editor

PLOS ONE

Journal Requirements:

When submitting your revision, we need you to address these additional requirements.

1. Please ensure that your manuscript meets PLOS ONE's style requirements, including those for file naming. The PLOS ONE style templates can be found at 

https://journals.plos.org/plosone/s/file?id=wjVg/PLOSOne_formatting_sample_main_body.pdf and 

https://journals.plos.org/plosone/s/file?id=ba62/PLOSOne_formatting_sample_title_authors_affiliations.pdf

2. Thank you for stating the following in the Acknowledgments Section of your manuscript: 

    "This work was supported by SPIRITS（Supporting Program for InteRaction-based Initiative Team Studies）2022 of Kyoto University"

We note that you have provided funding information that is not currently declared in your Funding Statement. However, funding information should not appear in the Acknowledgments section or other areas of your manuscript. We will only publish funding information present in the Funding Statement section of the online submission form. 

Please remove any funding-related text from the manuscript and let us know how you would like to update your Funding Statement. Currently, your Funding Statement reads as follows: 

   "This work was supported by SPIRITS（Supporting Program for InteRaction-based Initiative Team Studies）2022 of Kyoto University."

Please include your amended statements within your cover letter; we will change the online submission form on your behalf.

3. Thank you for stating the following financial disclosure: 

   "This work was supported by SPIRITS（Supporting Program for InteRaction-based Initiative Team Studies）2022 of Kyoto University."  

Please state what role the funders took in the study.  If the funders had no role, please state: "The funders had no role in study design, data collection and analysis, decision to publish, or preparation of the manuscript." 

If this statement is not correct you must amend it as needed. 

Please include this amended Role of Funder statement in your cover letter; we will change the online submission form on your behalf.

4. We note that you have stated that you will provide repository information for your data at acceptance. Should your manuscript be accepted for publication, we will hold it until you provide the relevant accession numbers or DOIs necessary to access your data. If you wish to make changes to your Data Availability statement, please describe these changes in your cover letter and we will update your Data Availability statement to reflect the information you provide.

5. Please include a copy of Table 1-5 which you refer to in your text on pages 10-11.

[Note: HTML markup is below. Please do not edit.]

Reviewers' comments:

Reviewer's Responses to Questions

**Comments to the Author**

1. Is the manuscript technically sound, and do the data support the conclusions?

The manuscript must describe a technically sound piece of scientific research with data that supports the conclusions. Experiments must have been conducted rigorously, with appropriate controls, replication, and sample sizes. The conclusions must be drawn appropriately based on the data presented. 

Reviewer #1: Partly

Reviewer #2: Partly

Reviewer #3: Partly

**********

2. Has the statistical analysis been performed appropriately and rigorously? 

Reviewer #1: No

Reviewer #2: N/A

Reviewer #3: N/A

**********

3. Have the authors made all data underlying the findings in their manuscript fully available?

The PLOS Data policy requires authors to make all data underlying the findings described in their manuscript fully available without restriction, with rare exception (please refer to the Data Availability Statement in the manuscript PDF file). The data should be provided as part of the manuscript or its supporting information, or deposited to a public repository. For example, in addition to summary statistics, the data points behind means, medians and variance measures should be available. If there are restrictions on publicly sharing data—e.g. participant privacy or use of data from a third party—those must be specified.

Reviewer #1: Yes

Reviewer #2: Yes

Reviewer #3: Yes

**********

4. Is the manuscript presented in an intelligible fashion and written in standard English?

PLOS ONE does not copyedit accepted manuscripts, so the language in submitted articles must be clear, correct, and unambiguous. Any typographical or grammatical errors should be corrected at revision, so please note any specific errors here.

Reviewer #1: No

Reviewer #2: No

Reviewer #3: No

**********

5. Review Comments to the Author

Please use the space provided to explain your answers to the questions above. You may also include additional comments for the author, including concerns about dual publication, research ethics, or publication ethics. (Please upload your review as an attachment if it exceeds 20,000 characters)

Reviewer #1: I would like to thank the authors for this work. The use of artificial intelligence and machine learning in health, research and education raises a lot of ethical concerns. Overall, certain aspects of the manuscript are difficult to follow because of the numerous grammatical errors and inappropriate use of tenses. This paper will benefit from editing by a native English speaker or scientific writer. Below are the detailed comments:

1. Page 2 Line 39: The authors referred to Belmont Report and the Declaration of Helsinki as “universal indicators” which I think is not accurate. These are more of international regulations than indicators. This should be revised.

2. The authors use the term "Joint research" which I suggest should be revised to "collaborative research".

3. Page 4 lines70-72: This is a factual statement that should be referenced.

4. Line 74-75: "There are many reports on the ethics of the use of AI in research [11] [12],

5. 75 but there is no report on how AI handles research ethics." What do the authors mean by "how AI handles research ethics?" That phrase does not seem to make a lot of sense.

6. The objective of the study is not concise and is poorly stated, as such, this makes it difficult to appreciate the authors arguments and line of thought. The objective should be recast.

7. The authors have outlined the questions they asked ChatGPT but they have not described how the data was analyzed. This aspect should be added to the methods section.

8. Line 228-231. Who was concerned? The authors or ChatGPT.

Discussion

9. Overall, the discussion is written in poor English and difficult to follow. It is difficult to discern what message the authors would like to communicate to the reader. The discussion section is majorly a recap of the results with minimal actual discussion and interpretation of the results.

10. The description of the ChatGPT output in the discussion is poorly done and confusing. In some instances, it is difficult to differentiate between the authors opinions and the study findings. Further, there is inappropriate use of "it'", "we" and "they".

11. Line 254-257: "Taken together, these findings revealed that ChatGPT understood the ethical principles and can provide the most efficient answers to various questions. On the other hand, there were cases where the understanding was extreme, resulting in answers that focused on one part of ethical considerations or answers that were shallow overall". What is meant by efficient answers? What is meant by extreme understanding? There are two conflicting messages in the above statement. I suggest that more elaboration is given. The discussion should be improved.

Conclusion

12. The authors stated that "In this study, an existing interactive AI was used to examine possible issues related to subject protection in research". But this is different from the objective stated in the introduction. Authors also conclude stated that "We discovered the usefulness and challenges of using AI to assist in ethical review". However, these are not reported in the result section. Furthermore, the discussion is shallow on these aspects. The objective of the study should be consistent and in line with the conclusion.

Reviewer #2: Unfortunately I recommend rejection for this manuscript even though I think it was a great idea for a study which could potentially be very interesting. But as currently described and constructed it wasn't particularly well implemented. What I think it has shown is that the AI can identify key principles from some notable policies, and also potential risks/side effects from certain clinical procedures, but I am not sure what this has told us about the process (or potential of AI) of/in ethics review.

Although the intention was to initially establish the foundations of an ethics review by referring to the declaration of Helsinki and Belmont report in particular, the study 1) did not spend enough time/detail defining the content of a "good" research ethics review for subsequent comparison with the AI ethics review. Linked to this, 2) research ethics committees review voluminous, complex, information about a project, whereas this study only presented extremely short vignettes to the AI. Unfortunately this makes the current work of little relevance to "real" ethics reviews where the difficulty almost always lies in the contextual details of projects under review. As this study did not address this point in particular, I think it has missed the key thing that AI might be able to add - the ability to rapidly review large and complex study protocols and identify the key issues. Rather than provide short vignettes, the researchers really needed to give ChatGPT some extensive study documentation and then see whether it could analyse it rapidly and accurately, identifying relevant ethical issues.

Furthermore, this manuscript needs 3) substantial proof reading and editing. For instance, the introduction needs a major revision to more accurately reflect the current situation within research ethics reviews. There are also inaccuracies such as the World Health Organisation definitely does not "assure the quality of ethics reviews", and while the examples of accreditation are interesting, there is a substantial literature about ethics committee consistency that has not been referenced. Likewise there are many guidance documents that list the questions that ethics committees need to ask when reviewing projects - it would be nice to have seen at least some of these referenced and perhaps the questions listed in a table/figure (going some ways to address my concern number 1, stated above).

Finally in a number of places there are statements about ChatGPT "understanding" ethical principles. I would question this language. Maybe it identified principles, but understanding is a very different (and difficult to define) issue.

Reviewer #3: This is an interesting article especially since it is one of the first, if not the first, to review ChatGPT's capacity to provide inputs to research ethics review. This makes the manuscript interesting and relevant. That said, before the manuscript would need a major and some minor revisions.

Major: the major issue of the article has to do with the fact that the methodology used to judge the quality of ChatGPT's responses is not identified in the methodology section nor made explicit in the succeeding sections. As it is, the reader is left guessing whether ChatGPT's responses were pitted against those of the researchers. If indeed so, how sufficient is this methodology for evaluation considering that the point of comparison should be research ethics committee members and their review processes?

There were also some minor issues that need to be addressed:

1. line 43-45: "The assurance of the quality of ethics committee reviews has long been carried out by the World Health Organization, an international human subject protection program." -- WHO is not an international human subject protection program, though this might be a grammatical issue.

2. line 54-54: "it is considered most important for the members of each committee 'to provide many opinions for subject protection'" -- The idea here is contestable. It is not the number of opinions ("provide many opinions) but whether the opinions are exhaustive to have captured all the relevant ethical concerns.

3. line 63-65: "However, in humanities and social sciences, because there are many ethics committees, the quality of

65 each committee is inconsistent, and the medical field is no exception" -- Can we realistically say that the number of ethics committees correlate to the quality of review?

4. line 65-67: Moreover, in joint research, the general rule is to conduct a collective review by asking the opinions of

one representative ethics committee,..." -- Please provide the context. Note that this is not a generalizable procedure.

5. line 69-70: "Therefore, it is important to have a common tool for each committee and a mechanism that allows a certain amount of opinions to be expressed" -- That a "common tool" is necessary is an assumption that needs some form of justification. Why are common standards not sufficient?

6. line 70-72: "Opinions expressed uniformly and homogeneously by natural language processing artificial intelligence (AI) are believed to contribute to improving the quality of ethics committees." -- Provide reference.

7. line 72-74: Furthermore, AI and machine learning were reported to prevent human errors in medical diagnosis and ethical review" [10] -- The reference cited did not include "ethical review", contrary to what the authors claim.

8. line 162-166: It seems that the researchers asked a question which was interpreted differently by ChatGPT. The researchers were probably asking about ChatGPT's knowledge of the "Japanese Ethical Guidelines for Medical and Health Research Involving Human Subjects," while ChatGPT understood the question to mean, "what are the ethical guidelines for medical and health research involving human subjects", i.e., a more generic question instead of the more specific question relating to ChatGPT's familiarity with the Japanese guideline. This raises the claims related to this answer as invalid.

9. In the Discussion section, was the word "we" used to refer to ChatGPT's responses?

**********

6. PLOS authors have the option to publish the peer review history of their article (what does this mean?). If published, this will include your full peer review and any attached files.

If you choose “no”, your identity will remain anonymous but your review may still be made public.

**Do you want your identity to be public for this peer review?** For information about this choice, including consent withdrawal, please see our Privacy Policy.

Reviewer #1: No

Reviewer #2: No

Reviewer #3: No

**********

[NOTE: If reviewer comments were submitted as an attachment file, they will be attached to this email and accessible via the submission site. Please log into your account, locate the manuscript record, and check for the action link "View Attachments". If this link does not appear, there are no attachment files.]

While revising your submission, please upload your figure files to the Preflight Analysis and Conversion Engine (PACE) digital diagnostic tool, https://pacev2.apexcovantage.com/. PACE helps ensure that figures meet PLOS requirements. To use PACE, you must first register as a user. Registration is free. Then, login and navigate to the UPLOAD tab, where you will find detailed instructions on how to use the tool. If you encounter any issues or have any questions when using PACE, please email PLOS at figures@plos.org. Please note that Supporting Information files do not need this step.

# Author comment: Author response to Decision Letter 0

**DOI:** 10.1371/journal.pone.0311148.r002

1 Aug 2023

Reviewer Comments 

→

Response

Reviewer １

1. Page 2 Line 39: The authors referred to Belmont Report and the Declaration of Helsinki as “universal indicators” which I think is not accurate. These are more of international regulations than indicators. This should be revised.

→

Thank you for pointing this out.

I revised it as follows.

universal indicators →　international regulations

2. The authors use the term "Joint research" which I suggest should be revised to "collaborative research".

→

Thank you for pointing this out.

I revised it as follows.

"Joint research" →"collaborative research"

3. Page 4 lines70-72: This is a factual statement that should be referenced. 

→

I corrected the article and referenced it.

4. Line 74-75: "There are many reports on the ethics of the use of AI in research [11] [12], 

→

I decided it was unnecessary and deleted it.

5. 75 but there is no report on how AI handles research ethics." What do the authors mean by "how AI handles research ethics?" That phrase does not seem to make a lot of sense. 

→I decided it was unnecessary and deleted it.

6. The objective of the study is not concise and is poorly stated, as such, this makes it difficult to appreciate the authors arguments and line of thought. The objective should be recast. 

→

Thank you for pointing this out.

I have revised the wording so that the purpose of this question is limited to "Thus, this study provided input summaries of various medical research into ChatGPT and examined whether interactive AI can discuss the ethical issues of research."

7. The authors have outlined the questions they asked ChatGPT but they have not described how the data was analyzed. This aspect should be added to the methods section. 

→

All the answers to the question were written down, a table was created, and the contents were examined by two researchers. I have added this information to the Methods.

8. Line 228-231. Who was concerned? The authors or ChatGPT. 

→

Shows the ChatGPT answer. In order to distinguish between the author and ChatGPT, the entire sentence has been corrected to use proper nouns.

9. Overall, the discussion is written in poor English and difficult to follow. It is difficult to discern what message the authors would like to communicate to the reader. The discussion section is majorly a recap of the results with minimal actual discussion and interpretation of the results. 

→

As much as possible, we have revised the discussion to make it easy to understand and reasonable.

10. The description of the ChatGPT output in the discussion is poorly done and confusing. In some instances, it is difficult to differentiate between the authors opinions and the study findings. Further, there is inappropriate use of "it'", "we" and "they". 

→

The output contents of ChatGPT have been clearly expressed in the text.

Specifically, I always used the subject of "ChatGPT" when answering ChatGPT.

11. Line 254-257: "Taken together, these findings revealed that ChatGPT understood the ethical principles and can provide the most efficient answers to various questions. On the other hand, there were cases where the understanding was extreme, resulting in answers that focused on one part of ethical considerations or answers that were shallow overall". What is meant by efficient answers? What is meant by extreme understanding? There are two conflicting messages in the above statement. I suggest that more elaboration is given. The discussion should be improved. 

→

English mistake. I fixed it as follows:

Taken together, these findings reveal that ChatGPT understands ethical principles and can provide the most basic ethical answers to various questions. In contrary, owing to the influence of machine learning, in some cases, responses were extreme, focused on some of the ethical considerations, or were generally shallow.

12. The authors stated that "In this study, an existing interactive AI was used to examine possible issues related to subject protection in research". But this is different from the objective stated in the introduction. Authors also conclude stated that "We discovered the usefulness and challenges of using AI to assist in ethical review". However, these are not reported in the result section. Furthermore, the discussion is shallow on these aspects. The objective of the study should be consistent and in line with the conclusion 

→

Thank you very much for your very important opinion. In the current situation, it was an expression that invites misunderstanding as a leap of logic. Changed the purpose at the beginning to whether or not to point out research ethics in generated AI. This time, I will state the results obtained with ChatGPT in the conclusion.

In other words, both the purpose and the conclusion have been revised.

Reviewer ２

Reviewer #2: Unfortunately I recommend rejection for this manuscript even though I think it was a great idea for a study which could potentially be very interesting. But as currently described and constructed it wasn't particularly well implemented. What I think it has shown is that the AI can identify key principles from some notable policies, and also potential risks/side effects from certain clinical procedures, but I am not sure what this has told us about the process (or potential of AI) of/in ethics review.

Although the intention was to initially establish the foundations of an ethics review by referring to the declaration of Helsinki and Belmont report in particular, the study 1) did not spend enough time/detail defining the content of a "good" research ethics review for subsequent comparison with the AI ethics review. Linked to this, 2) research ethics committees review voluminous, complex, information about a project, whereas this study only presented extremely short vignettes to the AI. Unfortunately this makes the current work of little relevance to "real" ethics reviews where the difficulty almost always lies in the contextual details of projects under review. As this study did not address this point in particular, I think it has missed the key thing that AI might be able to add - the ability to rapidly review large and complex study protocols and identify the key issues. Rather than provide short vignettes, the researchers really needed to give ChatGPT some extensive study documentation and then see whether it could analyse it rapidly and accurately, identifying relevant ethical issues.

Furthermore, this manuscript needs 3) substantial proof reading and editing. For instance, the introduction needs a major revision to more accurately reflect the current situation within research ethics reviews. There are also inaccuracies such as the World Health Organisation definitely does not "assure the quality of ethics reviews", and while the examples of accreditation are interesting, there is a substantial literature about ethics committee consistency that has not been referenced. Likewise there are many guidance documents that list the questions that ethics committees need to ask when reviewing projects - it would be nice to have seen at least some of these referenced and perhaps the questions listed in a table/figure (going some ways to address my concern number 1, stated above).

Finally in a number of places there are statements about ChatGPT "understanding" ethical principles. I would question this language. Maybe it identified principles, but understanding is a very different (and difficult to define) issue.

→

Thank you for your valuable opinion.

As you said, I understand that there is insufficient verification to compare with the Ethics Committee.

In this rivision paper, referring to this opinion and the opinions of other reviewers, we will focus on "Can we extract ethical opinions in research using generative AI?"

Significant revisions have been made to the portion of the current description showing a comparison with the actual Ethics Committee.

Added the question of whether AI understands ethical principles, which was a concern in the comments, to discussion of the study.

Reviewer ３

Major: the major issue of the article has to do with the fact that the methodology used to judge the quality of ChatGPT's responses is not identified in the methodology section nor made explicit in the succeeding sections. As it is, the reader is left guessing whether ChatGPT's responses were pitted against those of the researchers. If indeed so, how sufficient is this methodology for evaluation considering that the point of comparison should be research ethics committee members and their review processes? 

→

Thank you for your valuable feedback. Since it is not a level of verification that can be compared with direct ethical review, we have organized the purpose and conclusion and made significant revisions.

1. line 43-45: "The assurance of the quality of ethics committee reviews has long been carried out by the World Health Organization, an international human subject protection program." -- WHO is not an international human subject protection program, though this might be a grammatical issue. 

→

It was a grammatical error, so I fixed it as follows.

 In an effort to ensure the quality of Ethics Committee reviews, the World Health Organization has issued guidelines on review quality and practices.

2. line 54-54: "it is considered most important for the members of each committee 'to provide many opinions for subject protection'" -- The idea here is contestable. It is not the number of opinions ("provide many opinions) but whether the opinions are exhaustive to have captured all the relevant ethical concerns. 

→

As you said, I have modified it as follows.

On the other hand, to solve the quality problem of actual ethics committees, it is considered most important for the members of each committee to “"Provide a comprehensive opinion of ethical considerations for the protection of human subjects.".”

3. line 63-65: "However, in humanities and social sciences, because there are many ethics committees, the quality of

65 each committee is inconsistent, and the medical field is no exception" -- Can we realistically say that the number of ethics committees correlate to the quality of review? 

→

Sorry for the misunderstanding caused by my English mistake. We have corrected it as follows.

However, owing to the current situation in Japan, there are suspicions regarding the declining quality of ethics review in the humanities and social sciences owing to the disparity in opinions and review resources because of the excessive establishment of ethics committees.

4. line 65-67: Moreover, in joint research, the general rule is to conduct a collective review by asking the opinions of

one representative ethics committee,..." -- Please provide the context. Note that this is not a generalizable procedure.

→

I have inserted a paper and improved the description to show that the number of countries that have adopted collective review is increasing.

5. line 69-70: "Therefore, it is important to have a common tool for each committee and a mechanism that allows a certain amount of opinions to be expressed" -- That a "common tool" is necessary is an assumption that needs some form of justification. Why are common standards not sufficient? 

→

As you pointed out, there are various possible cases, so we have revised the description.

6. line 70-72: "Opinions expressed uniformly and homogeneously by natural language processing artificial intelligence (AI) are believed to contribute to improving the quality of ethics committees." -- Provide reference.

→

This is the opinion of the author, so I have revised it as follows.

Furthermore, AI and machine learning can reportedly prevent human errors in medical diagnosis [10]. An AI that detects such errors could be useful, for example, in enumerating ethical points from a research protocol.

7. line 72-74: Furthermore, AI and machine learning were reported to prevent human errors in medical diagnosis and ethical review" [10] -- The reference cited did not include "ethical review", contrary to what the authors claim.

→

As you pointed out, [10] does not include ethical review, so I deleted the "ethical review" part. 

I have added the following sentences.

--

An AI that detects such errors could be useful, for example, in enumerating ethical points from a research protocol.

8. line 162-166: It seems that the researchers asked a question which was interpreted differently by ChatGPT. The researchers were probably asking about ChatGPT's knowledge of the "Japanese Ethical Guidelines for Medical and Health Research Involving Human Subjects," while ChatGPT understood the question to mean, "what are the ethical guidelines for medical and health research involving human subjects", i.e., a more generic question instead of the more specific question relating to ChatGPT's familiarity with the Japanese guideline. This raises the claims related to this answer as invalid. 

→

We also asked whether or not they had studied, and received an answer that they had. I've added it to the Method section and Discussion to make that clear. Also, I added the following sentences so that it would not be an excessive expression.

--

However, it presently remains unclear whether it is possible to apply ethical principles to various research contents and respond flexibly about the topic of participant protection only with this understanding.

9. In the Discussion section, was the word "we" used to refer to ChatGPT's responses? 

→

I clarified the distinction between ChatGPT and the author.　Specifically, when referring to ChatGPT, I expressed it with a proper noun.

Attachment Submitted filename: Response to Reviewers.docx

# Peer review: Decision Letter 1

**DOI:** 10.1371/journal.pone.0311148.r003

**Authors:** Sally Mohammed Farghaly

22 Nov 2023

PONE-D-23-12275R1Can artificial intelligence point out the ethics of medical research?: Using a generative pretrained transformerPLOS ONE

Dear Dr. Takuya,

Thank you for submitting your manuscript to PLOS ONE. After careful consideration, we feel that it has merit but does not fully meet PLOS ONE’s publication criteria as it currently stands. Therefore, we invite you to submit a revised version of the manuscript that addresses the points raised during the review process.

Please submit your revised manuscript by Jan 06 2024 11:59PM. If you will need more time than this to complete your revisions, please reply to this message or contact the journal office at plosone@plos.org. When you're ready to submit your revision, log on to https://www.editorialmanager.com/pone/ and select the 'Submissions Needing Revision' folder to locate your manuscript file.

Please include the following items when submitting your revised manuscript:

- A rebuttal letter that responds to each point raised by the academic editor and reviewer(s). You should upload this letter as a separate file labeled 'Response to Reviewers'.
- A marked-up copy of your manuscript that highlights changes made to the original version. You should upload this as a separate file labeled 'Revised Manuscript with Track Changes'.
- An unmarked version of your revised paper without tracked changes. You should upload this as a separate file labeled 'Manuscript'.

If you would like to make changes to your financial disclosure, please include your updated statement in your cover letter. Guidelines for resubmitting your figure files are available below the reviewer comments at the end of this letter.

If applicable, we recommend that you deposit your laboratory protocols in protocols.io to enhance the reproducibility of your results. Protocols.io assigns your protocol its own identifier (DOI) so that it can be cited independently in the future. For instructions see: https://journals.plos.org/plosone/s/submission-guidelines#loc-laboratory-protocols. Additionally, PLOS ONE offers an option for publishing peer-reviewed Lab Protocol articles, which describe protocols hosted on protocols.io. Read more information on sharing protocols at https://plos.org/protocols?utm_medium=editorial-email&utm_source=authorletters&utm_campaign=protocols.

We look forward to receiving your revised manuscript.

Kind regards,

Sally Mohammed Farghaly

Academic Editor

PLOS ONE

[Note: HTML markup is below. Please do not edit.]

Reviewers' comments:

Reviewer's Responses to Questions

**Comments to the Author**

1. If the authors have adequately addressed your comments raised in a previous round of review and you feel that this manuscript is now acceptable for publication, you may indicate that here to bypass the “Comments to the Author” section, enter your conflict of interest statement in the “Confidential to Editor” section, and submit your "Accept" recommendation.

Reviewer #1: (No Response)

Reviewer #4: All comments have been addressed

**********

2. Is the manuscript technically sound, and do the data support the conclusions?

The manuscript must describe a technically sound piece of scientific research with data that supports the conclusions. Experiments must have been conducted rigorously, with appropriate controls, replication, and sample sizes. The conclusions must be drawn appropriately based on the data presented. 

Reviewer #1: No

Reviewer #4: Partly

**********

3. Has the statistical analysis been performed appropriately and rigorously? 

Reviewer #1: No

Reviewer #4: N/A

**********

4. Have the authors made all data underlying the findings in their manuscript fully available?

The PLOS Data policy requires authors to make all data underlying the findings described in their manuscript fully available without restriction, with rare exception (please refer to the Data Availability Statement in the manuscript PDF file). The data should be provided as part of the manuscript or its supporting information, or deposited to a public repository. For example, in addition to summary statistics, the data points behind means, medians and variance measures should be available. If there are restrictions on publicly sharing data—e.g. participant privacy or use of data from a third party—those must be specified.

Reviewer #1: No

Reviewer #4: Yes

**********

5. Is the manuscript presented in an intelligible fashion and written in standard English?

PLOS ONE does not copyedit accepted manuscripts, so the language in submitted articles must be clear, correct, and unambiguous. Any typographical or grammatical errors should be corrected at revision, so please note any specific errors here.

Reviewer #1: No

Reviewer #4: Yes

**********

6. Review Comments to the Author

Please use the space provided to explain your answers to the questions above. You may also include additional comments for the author, including concerns about dual publication, research ethics, or publication ethics. (Please upload your review as an attachment if it exceeds 20,000 characters)

Reviewer #1: 1. I have a problem with the title. "Ethics of medical research" is a bit vague. Do the authors mean ethical issue in medical research? Ethical considerations in medical research?

2. The abstract is not informative at all. For instance, the objective is poorly stated, the methods are missing, the primary results are not clear, and the conclusion is more of a recommendation. I suggest that the authors structure the abstract in the conventional way i.e., Introduction, Methods, Results and Conclusion.

3. Almost all reviewers felt that the introduction needed major revisions however the authors did not do so. The introduction does not flow well. In line 40, the authors mention the Belmont report and Declaration of Helsinki. In the next sentence they mention laws and regulations; however, these are not legal documents. They are ethical guidelines.

4. Line 46-47 "World Health Organization has issued guidelines on review quality and practices"; references for these guidelines should be cited.

5. All abbreviations should be written in full the first time they are used in the main body of the manuscript.

6. Line 51-52 "In Japan, the Ministry of Health, Labor and Welfare implemented an ethics committee certification system". But no elaboration is given, on reference is cited etc. The introduction is full of these isolated statements with no logical flow, as such, making it difficult to follow. The authors should avoid these isolated statements. More information should be provided on this system since this is a Japanese study.

7. Line 56-57: “Provide a comprehensive opinion of ethical considerations for the protection of human subjects.” Where was this quote obtained? The authors should provide a reference and page number.

8. Line 61-62: "In addition, in collaborative research involving multiple institutions, a single ethics committee is the mainstream for collective review". I do not understand this statement. Do the authors refer to a research ethics committee of record? Centralized ethics review boards for multi-centre studies? The reference cited is more than 10 years old. Do the recommendations of in that referenced study still hold?

9. Line 64 has a problem; it should be revised.

10. It seems reference 9 is in Japanese, this should be stated in the reference list.

11. Line 76-78: "Therefore, we aimed to investigate whether it is possible to extract ethical points in various research fields using existing interactive AI". I am not sure what "ethical points" the authors are referring to in this objective. The objective is not SMART and should thus be rephrased.

12. Line 90-91: "Thus, this study provided input summaries of various medical research into ChatGPT and examined whether interactive AI can discuss the ethical issues of research". Which of the two statements was the objective of the study?

13. Line 105-108: "In addition, as this study used AI to obtain answers of virtual questions, which were created with research content including ethical concerns considered by the author, and does not involve human participants, the need for approval from an ethics committee was waived". Does the above statement allude to ethical considerations for this study? If so, it should be revise by first stating that the study was exempted from further REC review because of A, B, C etc. The REC reference number for the waiver should be quoted.

14. Line 125-128: “Q2-1 A study of 50 dementia patients, we will analyze specific proteins in the blood of cognitive function tests and blood samples, and conduct research to confirm the correlation between cognitive function and specific proteins”. What are the ethical considerations required in this case. I think some of the questions that ChatGPT was asked did not make a lot of sense, unless there was a problem translating them to English. Do cognitive function tests have blood like it is stated in the above statement?

15. ChatGPT was asked these questions, but nothing is mentioned about how the responses were analysed. Reviewers requested for improvements in the methodology, but this was not satisfactorily done. The methods are still poorly described.

Results

16. From the way results are presented, in some instances, It is difficult to tell whether the results are from ChatGPT or are opinions of the authors. Result presentation should be improved.

17. Line 295: "Therefore, it was suggested that the opinion of ethical considerations, for which answers were streamlined by machine learning, lead to extreme answers." What are extreme answers?

18. The conclusion feels blunt and noncommittal. What are the implications of the study findings to practice, policy, and research?

19. What are the strengths and limitations of the study?

Reviewer #4: Abstract:

• I believe that the abstract can be further improved. Ensure the abstract concisely summarizes the key points of the study, including objectives, methods, results, and conclusions.

Introduction:

• The introduction does a good job establishing the rationale for exploring AI in ethics reviews. To take it a step further, provide more specifics on the documented quality issues with committee reviews (e.g. statistics on decreasing thoroughness, common oversights, etc). This will further strengthen the justification.

• When introducing ChatGPT, give 1-2 sentences on the basics of how it was developed/trained and why it represents a more advanced AI system. This context will help readers new to ChatGPT.

• Expand on the background more to provide a more comprehensive context for the study.

Methods:

• Clarify the rationale behind choosing the specific version of ChatGPT used in the study.

• Expand on the data analysis process. Specifically touch on:

� How were ChatGPT's responses evaluated for accuracy, depth, and ethicality? What metrics were used?

� Did the two experts review the responses independently first and then come together?

� Was there a standardized rating scale or an open-ended discussion?

� How were differences in opinion between the experts reconciled?

Discussion:

• Enhance the discussion of implications, limitations, and potential applications of AI in ethics reviews.

• Address the variability in ChatGPT's responses and discuss how this impacts its potential utility in ethical reviews.

Conclusion:

• Include recommendations for future research and potential improvements in AI for ethical reviews.

Furthermore:

• Throughout the manuscript, attention to grammatical precision and clarity is needed.

• The manuscript would benefit from thorough proofreading to correct minor language errors and enhance readability.

**********

7. PLOS authors have the option to publish the peer review history of their article (what does this mean?). If published, this will include your full peer review and any attached files.

If you choose “no”, your identity will remain anonymous but your review may still be made public.

**Do you want your identity to be public for this peer review?** For information about this choice, including consent withdrawal, please see our Privacy Policy.

Reviewer #1: No

Reviewer #4: No

**********

[NOTE: If reviewer comments were submitted as an attachment file, they will be attached to this email and accessible via the submission site. Please log into your account, locate the manuscript record, and check for the action link "View Attachments". If this link does not appear, there are no attachment files.]

While revising your submission, please upload your figure files to the Preflight Analysis and Conversion Engine (PACE) digital diagnostic tool, https://pacev2.apexcovantage.com/. PACE helps ensure that figures meet PLOS requirements. To use PACE, you must first register as a user. Registration is free. Then, login and navigate to the UPLOAD tab, where you will find detailed instructions on how to use the tool. If you encounter any issues or have any questions when using PACE, please email PLOS at figures@plos.org. Please note that Supporting Information files do not need this step.

# Author comment: Author response to Decision Letter 1

**DOI:** 10.1371/journal.pone.0311148.r004

21 Jan 2024

Dear Dr. Emily Chenette,

Thank you for your ongoing consideration of our manuscript for publication in PLOS ONE. We appreciate the time spent by you and the reviewers and believe the revised manuscript is improved. We have made extensive and sincere revisions to the manuscript.

Below we discuss responses to reviewer comments.

Reviewer＃１

 1. I have a problem with the title. "Ethics of medical research" is a bit vague. Do the authors mean ethical issue in medical research? Ethical considerations in medical research?

→

Thank you for pointing this out.I revised it as follows.

"Ethics of medical research"→”ethical considerations in medical research”

2. The abstract is not informative at all. For instance, the objective is poorly stated, the methods are missing, the primary results are not clear, and the conclusion is more of a recommendation. I suggest that the authors structure the abstract in the conventional way i.e., Introduction, Methods, Results and Conclusion.

→

Thank you for your important comments. As suggested, we have reorganized the article into an introduction, methods, results, and conclusion. In addition, as a result of reconsideration, we have made significant changes to the entire text.

3. Almost all reviewers felt that the introduction needed major revisions however the authors did not do so. The introduction does not flow well. In line 40, the authors mention the Belmont report and Declaration of Helsinki. In the next sentence they mention laws and regulations; however, these are not legal documents. They are ethical guidelines.

→

Thank you for your important feedback. An error occurred during English proofreading, and the words "laws and regulations" were used multiple times. We have reviewed the relevant sections and revised the ethical guidelines.

4. Line 46-47 "World Health Organization has issued guidelines on review quality and practices"; references for these guidelines should be cited.

→

Since the description was insufficient, we have provided a detailed description and cited the guidelines as a reference.

5. All abbreviations should be written in full the first time they are used in the main body of the manuscript.

→

We reviewed the manuscript and ensured that if we used an abbreviation, we wrote the full word first.

6. Line 51-52 "In Japan, the Ministry of Health, Labor and Welfare implemented an ethics committee certification system". But no elaboration is given, on reference is cited etc. The introduction is full of these isolated statements with no logical flow, as such, making it difficult to follow. The authors should avoid these isolated statements. More information should be provided on this system since this is a Japanese study.

→

Added information and reference URL for this project as a citation. I created a connection with the previous sentence to avoid becoming an orphan sentence.

7. Line 56-57: “Provide a comprehensive opinion of ethical considerations for the protection of human subjects.” Where was this quote obtained? The authors should provide a reference and page number.

→

Added reference papers. For a long time, the opinions of committee members have been considered important, and for example, in the field of genetics, there are many opinions regarding considerations regarding genetics, but there are no opinions regarding pain associated with testing, etc., which has been raised as a problem in papers. Furthermore, in the study of single IRBs, there are doubts that the quality of the committee members influences the review, and the opinions of the committee members are treated as an important factor in the quality of the review. I've added those papers to my bibliography.

 8. Line 61-62: "In addition, in collaborative research involving multiple institutions, a single ethics committee is the mainstream for collective review". I do not understand this statement. Do the authors refer to a research ethics committee of record? Centralized ethics review boards for multi-centre studies? The reference cited is more than 10 years old. Do the recommendations of in that referenced study still hold?

→Research funded by American NIH grants and research that complies with Japan's ethical guidelines are subject to single IRB review. In Japan, from 2022 onwards, all examinations will be single IRB review. To avoid misunderstandings, I have referred to some countries, including Japan, as it appears to be shared worldwide. Also, as you pointed out, the Single IRB has been controversial and is still being debated to ensure a more friendly Single IRB review. I have also added the advantages and disadvantages along with the cited references.

 9. Line 64 has a problem; it should be revised.

→

Fixed the duplicate parts. I am terribly sorry.

10. It seems reference 9 is in Japanese, this should be stated in the reference list.

→

Thank you for your confirmation. Added to reference list.

11. Line 76-78: "Therefore, we aimed to investigate whether it is possible to extract ethical points in various research fields using existing interactive AI". I am not sure what "ethical points" the authors are referring to in this objective. The objective is not SMART and should thus be rephrased.

→

In the introduction, we have improved the flow of sentences up to stating the purpose. Specifically, "Deterioration in the quality of IRB reviews" → "Past efforts to improve quality" → "Need to diversify committee members' opinions" → "Past advances in generative AI" → We have organized the overall flow of "Can AI point out the ethical considerations in medical research?" Based on the above, I have briefly stated the purpose in the last line.

12. Line 90-91: "Thus, this study provided input summaries of various medical research into ChatGPT and examined whether interactive AI can discuss the ethical issues of research". Which of the two statements was the objective of the study?

→

I deleted it because it was ambiguous. As per answer 11, the purpose section has been extensively updated.

13. Line 105-108: "In addition, as this study used AI to obtain answers of virtual questions, which were created with research content including ethical concerns considered by the author, and does not involve human participants, the need for approval from an ethics committee was waived". Does the above statement allude to ethical considerations for this study? If so, it should be revise by first stating that the study was exempted from further REC review because of A, B, C etc. The REC reference number for the waiver should be quoted.

→

It is also possible that the misunderstanding occurred due to an issue with English proofreading. As you pointed out, we have amended the article to include the reasons for exemption from ethics review. We have confirmed that there is no exemption number for cases that are not covered by domestic ethical guidelines and do not require ethical review, as our university's Ethics Committee does not issue a certificate of exemption from ethical review.

14. Line 125-128: “Q2-1 A study of 50 dementia patients, we will analyze specific proteins in the blood of cognitive function tests and blood samples, and conduct research to confirm the correlation between cognitive function and specific proteins”. What are the ethical considerations required in this case. I think some of the questions that ChatGPT was asked did not make a lot of sense, unless there was a problem translating them to English. Do cognitive function tests have blood like it is stated in the above statement?

→

Thank you for your important feedback. In this case, I adopted this question because if I use a blood sample, I want to consider whether it is a blood sample or an existing sample. Although the wording is ambiguous, we would like to use the answers to such questions as part of the results, so we will use this question.

15. ChatGPT was asked these questions, but nothing is mentioned about how the responses were analysed. Reviewers requested for improvements in the methodology, but this was not satisfactorily done. The methods are still poorly described.

→

Thank you for your frequent comments. We have received specific opinions from other reviewers, so we have updated the content. In addition, matters that could not be elucidated using the methods of this study were also noted in the discussion section as limitations of this study.

Results

16. From the way results are presented, in some instances, It is difficult to tell whether the results are from ChatGPT or are opinions of the authors. Result presentation should be improved.

→

Thank you for pointing this out. I changed the description to make it clear that this is ChatGPT's answer.

17. Line 295: "Therefore, it was suggested that the opinion of ethical considerations, for which answers were streamlined by machine learning, lead to extreme answers." What are extreme answers?

→

An "extreme answer" indicates that it is not a comprehensive opinion, but is limited to a few opinions. I added words to make it clearer.

18. The conclusion feels blunt and noncommittal. What are the implications of the study findings to practice, policy, and research?

→

Thank you for pointing this out. Added a description of how the research results will affect practice, policy, and research. We have revised it significantly.

19. What are the strengths and limitations of the study?

→

Added text to the manuscript regarding the limitations of this study. The breaking point is that ChatGPT's system evaluation is not rigorous, so it should be treated as just a ChatGPT answer. Furthermore, since the number of sentences in this question is small, it is unclear whether it can be applied to an actual research plan level. The advantage is that this is an unprecedented effort, and generative AI could be a useful tool for considering partial ethical issues.

Reviewer #4

Abstract:

• I believe that the abstract can be further improved. Ensure the abstract concisely summarizes the key points of the study, including objectives, methods, results, and conclusions.

→

Thank you for your important comments. As suggested, we have reorganized the article into an introduction, methods, results, and conclusion. In addition, as a result of reconsideration, we have made significant changes to the entire text.

Introduction:

• The introduction does a good job establishing the rationale for exploring AI in ethics reviews. To take it a step further, provide more specifics on the documented quality issues with committee reviews (e.g. statistics on decreasing thoroughness, common oversights, etc). This will further strengthen the justification.

• When introducing ChatGPT, give 1-2 sentences on the basics of how it was developed/trained and why it represents a more advanced AI system. This context will help readers new to ChatGPT.

• Expand on the background more to provide a more comprehensive context for the study.

→

In the introduction, we have improved the flow of sentences up to stating the purpose. Specifically, "Deterioration in the quality of IRB reviews" → "Past efforts to improve quality" → "Need to diversify committee members' opinions" → "Past advances in generative AI" → We have organized the overall flow of "Can AI point out the ethical considerations in medical research?" 

We have prepared a description of how to use ChatGPT that we believe can be applied to medical research by expanding the background and improving the description. Regarding issues related to the quality of the committee's review (oversight, lack of thoroughness), there is little objectively disclosed data, so we have organized the text to make it clear that the opinions of the committee members are more important.

Methods:

• Clarify the rationale behind choosing the specific version of ChatGPT used in the study.

• Expand on the data analysis process. Specifically touch on:

・ How were ChatGPT's responses evaluated for accuracy, depth, and ethicality? What metrics were used?

・ Did the two experts review the responses independently first and then come together?

・ Was there a standardized rating scale or an open-ended discussion?

・How were differences in opinion between the experts reconciled?

→

Added reason for version selection. Added description regarding data accuracy evaluation and analysis. It added that an open-ended discussion was held to conduct exploratory research.

Additionally, although discussions were held, it was not possible to examine the validity of ChatGPT in detail, which was added as a limitation of this study.

Discussion:

• Enhance the discussion of implications, limitations, and potential applications of AI in ethics reviews.

• Address the variability in ChatGPT's responses and discuss how this impacts its potential utility in ethical reviews.

→

We have added the limitations of this study to the discussion. Furthermore, given its limitations, we enumerated the potential usefulness of generative AI in reconsidering ethical issues in research.

Conclusion:

• Include recommendations for future research and potential improvements in AI for ethical reviews.

→

The conclusion section has been significantly revised to incorporate your comments.

Furthermore:

• Throughout the manuscript, attention to grammatical precision and clarity is needed.

• The manuscript would benefit from thorough proofreading to correct minor language errors and enhance readability.

→

Thank you for your feedback.

Both the first edition and the revision were proofread by an English proofreading company, but we apologize for not being able to provide a sufficient level of English. Once again, we outsourced the work to a proofreading company and requested a thorough English proofreading.

Attachment Submitted filename: Response to Reviewers.docx

# Peer review: Decision Letter 2

**DOI:** 10.1371/journal.pone.0311148.r005

**Authors:** Sally Mohammed Farghaly

8 Mar 2024

PONE-D-23-12275R2Can artificial intelligence point out the ethical considerations in medical research? Utilizing a generative pretrained transformerPLOS ONE

Dear Dr. Takuya,

Thank you for submitting your manuscript to PLOS ONE. After careful consideration, we feel that it has merit but does not fully meet PLOS ONE’s publication criteria as it currently stands. Therefore, we invite you to submit a revised version of the manuscript that addresses the points raised during the review process.

Please submit your revised manuscript by Apr 22 2024 11:59PM. If you will need more time than this to complete your revisions, please reply to this message or contact the journal office at plosone@plos.org. When you're ready to submit your revision, log on to https://www.editorialmanager.com/pone/ and select the 'Submissions Needing Revision' folder to locate your manuscript file.

Please include the following items when submitting your revised manuscript:

- A rebuttal letter that responds to each point raised by the academic editor and reviewer(s). You should upload this letter as a separate file labeled 'Response to Reviewers'.
- A marked-up copy of your manuscript that highlights changes made to the original version. You should upload this as a separate file labeled 'Revised Manuscript with Track Changes'.
- An unmarked version of your revised paper without tracked changes. You should upload this as a separate file labeled 'Manuscript'.

If you would like to make changes to your financial disclosure, please include your updated statement in your cover letter. Guidelines for resubmitting your figure files are available below the reviewer comments at the end of this letter.

If applicable, we recommend that you deposit your laboratory protocols in protocols.io to enhance the reproducibility of your results. Protocols.io assigns your protocol its own identifier (DOI) so that it can be cited independently in the future. For instructions see: https://journals.plos.org/plosone/s/submission-guidelines#loc-laboratory-protocols. Additionally, PLOS ONE offers an option for publishing peer-reviewed Lab Protocol articles, which describe protocols hosted on protocols.io. Read more information on sharing protocols at https://plos.org/protocols?utm_medium=editorial-email&utm_source=authorletters&utm_campaign=protocols.

We look forward to receiving your revised manuscript.

Kind regards,

Sally Mohammed Farghaly

Academic Editor

PLOS ONE

Journal Requirements:

Please review your reference list to ensure that it is complete and correct. If you have cited papers that have been retracted, please include the rationale for doing so in the manuscript text, or remove these references and replace them with relevant current references. Any changes to the reference list should be mentioned in the rebuttal letter that accompanies your revised manuscript. If you need to cite a retracted article, indicate the article’s retracted status in the References list and also include a citation and full reference for the retraction notice.

[Note: HTML markup is below. Please do not edit.]

Reviewers' comments:

Reviewer's Responses to Questions

**Comments to the Author**

1. If the authors have adequately addressed your comments raised in a previous round of review and you feel that this manuscript is now acceptable for publication, you may indicate that here to bypass the “Comments to the Author” section, enter your conflict of interest statement in the “Confidential to Editor” section, and submit your "Accept" recommendation.

Reviewer #5: All comments have been addressed

Reviewer #6: (No Response)

Reviewer #7: All comments have been addressed

Reviewer #8: (No Response)

**********

2. Is the manuscript technically sound, and do the data support the conclusions?

The manuscript must describe a technically sound piece of scientific research with data that supports the conclusions. Experiments must have been conducted rigorously, with appropriate controls, replication, and sample sizes. The conclusions must be drawn appropriately based on the data presented. 

Reviewer #5: Yes

Reviewer #6: Partly

Reviewer #7: Yes

Reviewer #8: No

**********

3. Has the statistical analysis been performed appropriately and rigorously? 

Reviewer #5: N/A

Reviewer #6: N/A

Reviewer #7: N/A

Reviewer #8: N/A

**********

4. Have the authors made all data underlying the findings in their manuscript fully available?

The PLOS Data policy requires authors to make all data underlying the findings described in their manuscript fully available without restriction, with rare exception (please refer to the Data Availability Statement in the manuscript PDF file). The data should be provided as part of the manuscript or its supporting information, or deposited to a public repository. For example, in addition to summary statistics, the data points behind means, medians and variance measures should be available. If there are restrictions on publicly sharing data—e.g. participant privacy or use of data from a third party—those must be specified.

Reviewer #5: Yes

Reviewer #6: Yes

Reviewer #7: Yes

Reviewer #8: No

**********

5. Is the manuscript presented in an intelligible fashion and written in standard English?

PLOS ONE does not copyedit accepted manuscripts, so the language in submitted articles must be clear, correct, and unambiguous. Any typographical or grammatical errors should be corrected at revision, so please note any specific errors here.

Reviewer #5: Yes

Reviewer #6: No

Reviewer #7: Yes

Reviewer #8: No

**********

6. Review Comments to the Author

Please use the space provided to explain your answers to the questions above. You may also include additional comments for the author, including concerns about dual publication, research ethics, or publication ethics. (Please upload your review as an attachment if it exceeds 20,000 characters)

Reviewer #5: I was invited to review the manuscript after the second round of reviews and will try to be as concise as possible.

The topic is very up-to-date and there are some new papers on ChatGPT and bioethics. The article could be placed somewhat more in context of the discussion by adding additional references. The issue of The American Journal of Bioethics, Volume 23, 2023 - Issue 10 could be helpful, for instance. If these papers that were published (I guess) after submitting the manuscript have too much of an effect on the central argument they can still be noted as issues in need for further research.

While not referring to ChatGPT, there is an interesting paper that relates to this topic:

Lukas J. Meier, Alice Hein, Klaus Diepold & Alena Buyx (2022) Algorithms for Ethical Decision-Making in the Clinic: A Proof of Concept, The American Journal of Bioethics, 22:7, 4-20, DOI: 10.1080/15265161.2022.2040647

Here the authors drop the “justice” element, as it was “too difficult” to include. It appears that AI models are better at applying some principles (especially informed consent) than others. This fits well with the discussion by the authors.

Furthermore, there is a discussion in applied ethics stating that ethics should be more than a mere tick box exercise. While it is useful to have tick boxes to avoid missing standard issues, they do not invite to weight different factors. Much of the discussion refers to this problem and it would be important to put the discussion in light of this older debate on doing applied ethics (or methods in applied ethics).

Reviewer #6: Thank you for the opportunity to review this article!

The authors present an interesting and timely research idea. No doubt that the potential use of AI in research ethics review process will be (and already is) intensively discussed.

Nevertheless, the research study and the paper include significant shortcomings as already pointed out by previous reviewers. First and foremost, the methodology of the study is rather vague as has been pointed out by Reviewer 1. The data in the appendix show that the authors have used very short fictional summaries of research studies (one sentence) to inquire ChatGPT about ethical issues in these scenarios/questions. It is not clear whether these scenarios/questions were originally written in Japanese or English and what was the language of conversation with ChatGPT. In any case, this process is not comparable to a real ethics review where REC members are reviewing the protocol, informed consent form and other documents in full. In my view, to make any conclusions about the capacity of AI to discuss ethical issues in research protocols, the study should be built on real documents or at least fictional research protocols and informed consent forms at full length. Additionally, the scenarios/questions as they are presented in the appendix include mistakes and ambiguities. It is also not justified why specifically ChatGPT was used for generating answers since there are many different AI tools available for generating texts. It is not clear how the results were analyzed. The only indication of the approach to analysis are the phrases “two research ethics experts confirmed and examined the contents” and “then engaged in open-ended discussions about the content”. To my knowledge, it is not a methodical approach to analysis. For strong and rigorous analysis much more detailed method of analysis has to be applied, and there are many approaches in qualitative analysis that might be successfully used.

I also concur with the previous reviewers that the introduction and discussion do not flow well and are rather fragmented. Unfortunately, the two attempts to improve the text following the reviewers' comments do not seem to be fully successful. I guess that one of the reasons for that is the shortcomings in the methodology mentioned above. Consequently, the conclusions are very vague and it is not clear how they are supported by the data.

My suggestion to the authors is to continue the research by developing more detailed research idea and aim, implementing a new research study and writing a new, much stronger paper.

Reviewer #7: Thank you for allowing me to review this manuscript. The topic is interesting and timely. Kindly find my few comments below.

• I think the title should be modified. The second part of the title is not complete “Utilizing a generative pretrained transformer” for what? It can be, for example “Utilizing a generative pretrained transformer to address this issue.”

• The same applies for the short title.

• Abstract

o The goal of the study was to explore the use of ChatGPT “to enumerate ethical issues in medical research.”. I am not sure if the word “enumerate” is the correct choice. Enumeration is related to counting or listing items, and I don’t think this is the case here. Maybe “discuss” or other word will be more suitable. Please check.

• Introduction

o The previous point applies also for the introduction, page 4, line 80.

o Page 4, line 83, the authors mentioned that “Developed by Open AI, ChatGPT is capable of challenging incorrect assumptions.” I think this statement should be modified, because the chatbot still has limitations regarding accuracy.

Reviewer #8: Abstract:

The manuscript presents an exploratory study on the use of generative AI, specifically ChatGPT, to enumerate ethical considerations in medical research. The authors aimed to assess the AI's ability to identify ethical issues in various research contexts, using the AI's responses to structured questions that simulate real-world research scenarios. While the innovative approach and attempt to address the challenges of ethical review in medical research is commendable, several major concerns and limitations warrant attention.

Major Concerns:

-The study shows that while ChatGPT can enumerate basic ethical considerations, it struggles with the depth and nuanced understanding required for comprehensive ethical analysis. This limitation is particularly troubling in complex cases or where subtlety in ethical reasoning is critical. AI's inability to provide specific measures for psychological considerations or broader ethical issues underscores this shortcoming.

-Also, why was GPT3.5 and now GPT4 chosen? IMO, this does not make much sense.

Bias and machine learning optimization:

-The manuscript acknowledges the potential for biased opinions due to machine learning optimization, but does not thoroughly explore or address how these biases might impact the ethical review process. Given the critical importance of unbiased ethical considerations in medical research, the potential for AI to perpetuate existing biases or introduce new ones is a significant concern.

Methodological limitations:

-The methodology relies heavily on the current capabilities of AI without a robust framework for evaluating the ethical -depth or relevance of its responses. The lack of comparative analysis with human ethics committee decisions or discussion of how AI-derived insights integrate with human judgment is a notable gap.

-A more critical examination of areas where ChatGPT falls short, especially in complex ethical scenarios, would provide a more balanced view of its applicability in ethical reviews.

-The discussion reiterates results without fully leveraging them to explore the broader implications for AI in ethical decision-making.

Practical implications and integration challenges:

-While the manuscript suggests potential applications of generative AI to support ethical review, it does not adequately address the practical challenges of integrating AI insights into existing ethical review frameworks. The operational, legal, and ethical implications of relying on AI for ethical considerations in medical research are not explored in depth.

Reproducibility and transparency:

-The reproducibility of the study is questionable given the evolving nature of AI models such as ChatGPT. Changes to the model's training data or algorithms could significantly alter its responses over time, affecting the study's relevance and applicability in future research contexts.

Minor:

-Discussion of variability in ethical guidelines: There is insufficient discussion of the variability of ethical guidelines across countries, and how this variability affects the AI's ability to provide universally applicable ethical insights.

Overall assessment:

While the study presents an innovative approach, the implications of the findings for practical application in the ethical review process are not convincingly demonstrated. Also, the manuscript seems to be written by GPT in large parts, indicated by GPT-typical phrases, e.g. "realm of".. Overall, I am not convinced of the scientific benefit of this work.

**********

7. PLOS authors have the option to publish the peer review history of their article (what does this mean?). If published, this will include your full peer review and any attached files.

If you choose “no”, your identity will remain anonymous but your review may still be made public.

**Do you want your identity to be public for this peer review?** For information about this choice, including consent withdrawal, please see our Privacy Policy.

Reviewer #5: No

Reviewer #6: No

Reviewer #7: **Yes: **Ahmed Abdelhafiz

Reviewer #8: No

**********

[NOTE: If reviewer comments were submitted as an attachment file, they will be attached to this email and accessible via the submission site. Please log into your account, locate the manuscript record, and check for the action link "View Attachments". If this link does not appear, there are no attachment files.]

While revising your submission, please upload your figure files to the Preflight Analysis and Conversion Engine (PACE) digital diagnostic tool, https://pacev2.apexcovantage.com/. PACE helps ensure that figures meet PLOS requirements. To use PACE, you must first register as a user. Registration is free. Then, login and navigate to the UPLOAD tab, where you will find detailed instructions on how to use the tool. If you encounter any issues or have any questions when using PACE, please email PLOS at figures@plos.org. Please note that Supporting Information files do not need this step.

# Author comment: Author response to Decision Letter 2

**DOI:** 10.1371/journal.pone.0311148.r006

19 Apr 2024

Dear Dr. Emily Chenette,

Thank you for your ongoing consideration of our manuscript for publication in PLOS ONE. We appreciate the time spent by you and the reviewers and believe the revised manuscript is improved. We have made extensive and sincere revisions to the manuscript.　Below we discuss responses to reviewer comments.

Reviewer＃5

→

Thank you very much for your review.

I learned a lot from the advice I received regarding the discussion.

I read the paper you presented and added it to the discussion section regarding the advantages and disadvantages of using AI for ethical decision making.

Other reviewers also raised concerns about the use of AI, which were limitations of this study, and added them to the discussion.

Thank you for understanding.

Reviewer #6:

→

Thank you very much for your review.

I learned a lot from the advice I received as a result of this review.

1) First and foremost, the methodology of the study is rather vague as has been pointed out by Reviewer 1. The data in the appendix show that the authors have used very short fictional summaries of research studies (one sentence) to inquire ChatGPT about ethical issues in these scenarios/questions. It is not clear whether these scenarios/questions were originally written in Japanese or English and what was the language of conversation with ChatGPT. 

→

Thank you for pointing out the methods section.

The methods section has been significantly revised.

First, the short sentences in question were written in English, taking into consideration the difficulty of processing long sentences in generative AI models and the fact that learning conditions differ depending on the language.

As you pointed out, what actually took place in this study was to discuss research ethics in a short dialogue format, rather than a review at the research plan level, so we reorganized the method text.

2) In any case, this process is not comparable to a real ethics review where REC members are reviewing the protocol, informed consent form and other documents in full. In my view, to make any conclusions about the capacity of AI to discuss ethical issues in research protocols, the study should be built on real documents or at least fictional research protocols and informed consent forms at full length. 

→

This research was merely an exploratory study looking at the answers of existing generative AI, and due to the performance of the generative AI, it was not possible to process actual research plans or explanatory documents.

Therefore, as you pointed out, it is far from the REC process.

I have added this point as a limitation of the research.

3) It is also not justified why specifically ChatGPT was used for generating answers since there are many different AI tools available for generating texts. 

→

Thank you for pointing this out.

We are aware that there are currently countless generative AIs around the world that implement LLM, but this paper was first submit in April 2023, and we have been conducting research since shortly after ChatGPT was released. Ta. In addition, the reason for choosing ChatGPT is that it is a generative AI that is used by many users, as well as having been published in academic papers and undergoing numerous verifications. I have included these as reasons for my selection.

4) It is not clear how the results were analyzed. The only indication of the approach to analysis are the phrases “two research ethics experts confirmed and examined the contents” and “then engaged in open-ended discussions about the content”. To my knowledge, it is not a methodical approach to analysis. For strong and rigorous analysis much more detailed method of analysis has to be applied, and there are many approaches in qualitative analysis that might be successfully used.

→

Thank you for pointing this out.

He added that the analysis method also considered consistency with existing papers.

Also, as you pointed out, this survey does not allow for qualitative analysis. The reason is that this is a challenging exploratory study, and we believe that only the answers of the current generation of AI need to be discussed immediately. However, the lack of qualitative analysis is an issue for this study, so we added it as a main research issue in the discussion section.

Reviewer #7

→

Thank you very much for your review.

I learned a lot from the advice I received as a result of this review.

• I think the title should be modified. The second part of the title is not complete “Utilizing a generative pretrained transformer” for what? It can be, for example “Utilizing a generative pretrained transformer to address this issue.”

• The same applies for the short title.

→

Thank you for your feedback.

We have corrected the title and short title according to your suggestions, so please check them.

• Abstract

o The goal of the study was to explore the use of ChatGPT “to enumerate ethical issues in medical research.”. I am not sure if the word “enumerate” is the correct choice. Enumeration is related to counting or listing items, and I don’t think this is the case here. Maybe “discuss” or other word will be more suitable. Please check.

→

As you pointed out, "discuss" was appropriate, so we changed it to "discuss".

• Introduction

o The previous point applies also for the introduction, page 4, line 80.

→

As you pointed out, "discuss" was appropriate, so we changed it to "discuss".

o Page 4, line 83, the authors mentioned that “Developed by Open AI, ChatGPT is capable of challenging incorrect assumptions.” I think this statement should be modified, because the chatbot still has limitations regarding accuracy.

→

Thank you for your important comments.

As you said, we have added to the text that there is still room for discussion regarding the system for generating AI responses.

Reviewer #8

→

Thank you very much for your review.

I learned a lot from the many comments that get to the heart of the matter.

As you have pointed out, this paper shows that, in a sense, it is difficult to discuss research ethics with current generative AI.

Please note that we do not promote the use of ChatGPT, but rather that we aim to quickly introduce the current level of generative AI to the world and hope for further development in the future.

-Also, why was GPT3.5 and now GPT4 chosen? IMO, this does not make much sense.

→

Thank you for pointing this out.

We are aware that there are currently countless generative AIs around the world that implement LLM, but this paper was first submit in April 2023, and we have been conducting research since shortly after ChatGPT was released. Ta. In addition, the reason for choosing ChatGPT is that it is a generative AI that is used by many users, as well as having been published in academic papers and undergoing numerous verifications. We also chose version 3.5 because it is widely available for free and has the largest number of end users among generative AIs. We have added the reasons for these choices to the methods section.

-The manuscript acknowledges the potential for biased opinions due to machine learning optimization, but does not thoroughly explore or address how these biases might impact the ethical review process. Given the critical importance of unbiased ethical considerations in medical research, the potential for AI to perpetuate existing biases or introduce new ones is a significant concern.

→

Thank you for your important comments.

In this case, as you pointed out, biases are also learned, so we have added a new note in the discussion section to note that there is a concern that the AI could state existing biases or create new ones. .

-The methodology relies heavily on the current capabilities of AI without a robust framework for evaluating the ethical -depth or relevance of its responses. The lack of comparative analysis with human ethics committee decisions or discussion of how AI-derived insights integrate with human judgment is a notable gap.

→

Thank you for your important comments.

In this case, there was a lack of a framework for considering the ethical depth and relevance of AI responses, so we have added this to the discussion section as a limitation of the research.

We have also added that it has not been possible to compare and consider human responses.

Since this is currently an exploratory study, we have decided to address this issue as a future issue.

-A more critical examination of areas where ChatGPT falls short, especially in complex ethical scenarios, would provide a more balanced view of its applicability in ethical reviews.

→

Thank you for your feedback.

In this revision, we have made significant additions to the effect that the use of LLM's generation AI needs to be proceeded with caution and that the current GPT answers need to be critically examined.

While the manuscript suggests potential applications of generative AI to support ethical review, it does not adequately address the practical challenges of integrating AI insights into existing ethical review frameworks. The operational, legal, and ethical implications of relying on AI for ethical considerations in medical research are not explored in depth.

→

Thank you for your important comments.

At this stage, we understand that actual operation is a long way off.

Also, as you point out, the operational, legal and ethical implications need to be considered.

We have added this to the discussion section as a limitation of this study.

-The reproducibility of the study is questionable given the evolving nature of AI models such as ChatGPT. Changes to the model's training data or algorithms could significantly alter its responses over time, affecting the study's relevance and applicability in future research contexts.

→

As you said, I added to the discussion section that the reproducibility of answers can change depending on the learning status of LMM, and that it is necessary to understand this and carefully consider the answers.

-Discussion of variability in ethical guidelines: There is insufficient discussion of the variability of ethical guidelines across countries, and how this variability affects the AI's ability to provide universally applicable ethical insights.

→

I have organized and added this as a future issue.

Attachment Submitted filename: Response to Reviewers.docx

# Peer review: Decision Letter 3

**DOI:** 10.1371/journal.pone.0311148.r007

**Authors:** Sally Mohammed Farghaly

27 May 2024

PONE-D-23-12275R3Exploring Ethical Considerations in Medical Research: Harnessing Pre-Generated Transformers for AI-Powered Ethics DiscussionsPLOS ONE

Dear Dr. Mori,

Thank you for submitting your manuscript to PLOS ONE. After careful consideration, we feel that it has merit but does not fully meet PLOS ONE’s publication criteria as it currently stands. Therefore, we invite you to submit a revised version of the manuscript that addresses the points raised during the review process.

Please submit your revised manuscript by Jul 11 2024 11:59PM. If you will need more time than this to complete your revisions, please reply to this message or contact the journal office at plosone@plos.org. When you're ready to submit your revision, log on to https://www.editorialmanager.com/pone/ and select the 'Submissions Needing Revision' folder to locate your manuscript file.

Please include the following items when submitting your revised manuscript:

- A rebuttal letter that responds to each point raised by the academic editor and reviewer(s). You should upload this letter as a separate file labeled 'Response to Reviewers'.
- A marked-up copy of your manuscript that highlights changes made to the original version. You should upload this as a separate file labeled 'Revised Manuscript with Track Changes'.
- An unmarked version of your revised paper without tracked changes. You should upload this as a separate file labeled 'Manuscript'.

If you would like to make changes to your financial disclosure, please include your updated statement in your cover letter. Guidelines for resubmitting your figure files are available below the reviewer comments at the end of this letter.

If applicable, we recommend that you deposit your laboratory protocols in protocols.io to enhance the reproducibility of your results. Protocols.io assigns your protocol its own identifier (DOI) so that it can be cited independently in the future. For instructions see: https://journals.plos.org/plosone/s/submission-guidelines#loc-laboratory-protocols. Additionally, PLOS ONE offers an option for publishing peer-reviewed Lab Protocol articles, which describe protocols hosted on protocols.io. Read more information on sharing protocols at https://plos.org/protocols?utm_medium=editorial-email&utm_source=authorletters&utm_campaign=protocols.

We look forward to receiving your revised manuscript.

Kind regards,

Sally Mohammed Farghaly

Academic Editor

PLOS ONE

Journal Requirements:

Please review your reference list to ensure that it is complete and correct. If you have cited papers that have been retracted, please include the rationale for doing so in the manuscript text, or remove these references and replace them with relevant current references. Any changes to the reference list should be mentioned in the rebuttal letter that accompanies your revised manuscript. If you need to cite a retracted article, indicate the article’s retracted status in the References list and also include a citation and full reference for the retraction notice.

[Note: HTML markup is below. Please do not edit.]

Reviewers' comments:

Reviewer's Responses to Questions

**Comments to the Author**

1. If the authors have adequately addressed your comments raised in a previous round of review and you feel that this manuscript is now acceptable for publication, you may indicate that here to bypass the “Comments to the Author” section, enter your conflict of interest statement in the “Confidential to Editor” section, and submit your "Accept" recommendation.

Reviewer #1: All comments have been addressed

Reviewer #6: (No Response)

**********

2. Is the manuscript technically sound, and do the data support the conclusions?

The manuscript must describe a technically sound piece of scientific research with data that supports the conclusions. Experiments must have been conducted rigorously, with appropriate controls, replication, and sample sizes. The conclusions must be drawn appropriately based on the data presented. 

Reviewer #1: Yes

Reviewer #6: Partly

**********

3. Has the statistical analysis been performed appropriately and rigorously? 

Reviewer #1: N/A

Reviewer #6: N/A

**********

4. Have the authors made all data underlying the findings in their manuscript fully available?

The PLOS Data policy requires authors to make all data underlying the findings described in their manuscript fully available without restriction, with rare exception (please refer to the Data Availability Statement in the manuscript PDF file). The data should be provided as part of the manuscript or its supporting information, or deposited to a public repository. For example, in addition to summary statistics, the data points behind means, medians and variance measures should be available. If there are restrictions on publicly sharing data—e.g. participant privacy or use of data from a third party—those must be specified.

Reviewer #1: Yes

Reviewer #6: Yes

**********

5. Is the manuscript presented in an intelligible fashion and written in standard English?

PLOS ONE does not copyedit accepted manuscripts, so the language in submitted articles must be clear, correct, and unambiguous. Any typographical or grammatical errors should be corrected at revision, so please note any specific errors here.

Reviewer #1: Yes

Reviewer #6: (No Response)

**********

6. Review Comments to the Author

Please use the space provided to explain your answers to the questions above. You may also include additional comments for the author, including concerns about dual publication, research ethics, or publication ethics. (Please upload your review as an attachment if it exceeds 20,000 characters)

Reviewer #1: Thanks for endeavoring to address the numerous comments from the reviewers. The utility of AI in research ethics review is questionable and more research is needed.

Reviewer #6: Thank you for the response to my comments!

As I noted in my previous review, the research idea presented by the authors is interesting and timely. Nevertheless, the methodology of the research study is vague, and the details added in this revision still do not allow a full understanding of the methods used for data analysis. For example, the sentence “Two research ethics experts then engaged in open-ended discussions about the content” cannot be viewed as a detailed description of the method of analysis.

Also, as I mentioned in the previous review, the scenarios/questions asked to ChatGPT are very short and are not comparable to a real ethics review. As a result, the ChatGPT answers are very short and uniform, for example, the paragraph about informed consent or vulnerability is almost identical for all scenarios. The authors have tried to analyse the ChatGPT responses as various individual responses for each case, however, it might be worth analysing the overlaps between the answers which might lead to the conclusion that the answers are constantly repeated.

Additionally, I still hold the view that the discussion and conclusions are vague. For example, the authors write in the discussion that “it remains unclear whether it can apply ethical principles to diverse research scenarios and respond flexibly to subject protection opinions”, meaning that additional research and most likely different methodology is needed to answer this question. The current version of the conclusion does not bring anything new to the scientific discussion. Why not do further research, generate more data, apply more rigorous methodology, publish more detailed results and meaningful conclusions? My suggestion to authors would be to develop this research idea and do additional research, especially taking into account the limitations of this study indicated by the authors.

**********

7. PLOS authors have the option to publish the peer review history of their article (what does this mean?). If published, this will include your full peer review and any attached files.

If you choose “no”, your identity will remain anonymous but your review may still be made public.

**Do you want your identity to be public for this peer review?** For information about this choice, including consent withdrawal, please see our Privacy Policy.

Reviewer #1: No

Reviewer #6: No

**********

[NOTE: If reviewer comments were submitted as an attachment file, they will be attached to this email and accessible via the submission site. Please log into your account, locate the manuscript record, and check for the action link "View Attachments". If this link does not appear, there are no attachment files.]

While revising your submission, please upload your figure files to the Preflight Analysis and Conversion Engine (PACE) digital diagnostic tool, https://pacev2.apexcovantage.com/. PACE helps ensure that figures meet PLOS requirements. To use PACE, you must first register as a user. Registration is free. Then, login and navigate to the UPLOAD tab, where you will find detailed instructions on how to use the tool. If you encounter any issues or have any questions when using PACE, please email PLOS at figures@plos.org. Please note that Supporting Information files do not need this step.

# Author comment: Author response to Decision Letter 3

**DOI:** 10.1371/journal.pone.0311148.r008

29 May 2024

Dear Dr. Emily Chenette,

Thank you for your ongoing consideration of our manuscript for publication in PLOS ONE. We appreciate the time spent by you and the reviewers and believe the revised manuscript is improved. We have made extensive and sincere revisions to the manuscript.

Below we discuss responses to reviewer comments.

Reviewer #1: Thanks for endeavoring to address the numerous comments from the reviewers. The utility of AI in research ethics review is questionable and more research is needed.

→Thank you very much for your understanding. We greatly appreciate your feedback on the limitations of our study. We will consider these points carefully in future research, aiming to collect more detailed data and apply more rigorous methodologies to address these issues.

Reviewer #6: Thank you for the response to my comments!

As I noted in my previous review, the research idea presented by the authors is interesting and timely. Nevertheless, the methodology of the research study is vague, and the details added in this revision still do not allow a full understanding of the methods used for data analysis. For example, the sentence “Two research ethics experts then engaged in open-ended discussions about the content” cannot be viewed as a detailed description of the method of analysis.

Also, as I mentioned in the previous review, the scenarios/questions asked to ChatGPT are very short and are not comparable to a real ethics review. As a result, the ChatGPT answers are very short and uniform, for example, the paragraph about informed consent or vulnerability is almost identical for all scenarios. The authors have tried to analyse the ChatGPT responses as various individual responses for each case, however, it might be worth analysing the overlaps between the answers which might lead to the conclusion that the answers are constantly repeated.

Additionally, I still hold the view that the discussion and conclusions are vague. For example, the authors write in the discussion that “it remains unclear whether it can apply ethical principles to diverse research scenarios and respond flexibly to subject protection opinions”, meaning that additional research and most likely different methodology is needed to answer this question. The current version of the conclusion does not bring anything new to the scientific discussion. Why not do further research, generate more data, apply more rigorous methodology, publish more detailed results and meaningful conclusions? My suggestion to authors would be to develop this research idea and do additional research, especially taking into account the limitations of this study indicated by the authors.

→

Thank you very much for your thoughtful and detailed feedback on our manuscript.

As you noted in your previous review, we appreciate your recognition of the interest and timeliness of our research idea. 

We understand and acknowledge your concerns regarding the vagueness of the research methodology and the insufficient details provided in our revision. We recognize that the sentence “Two research ethics experts then engaged in open-ended discussions about the content” does not provide a comprehensive description of the analysis method. We have addressed this by including discussions based on previously published studies to clarify the points discussed by the two researchers. In future revisions, we will aim to include a more detailed explanation of our methodology to enhance clarity and understanding.

Regarding the scenarios and questions posed to ChatGPT, we agree that they are relatively short and not fully comparable to a real ethics review. This limitation has resulted in ChatGPT's responses being very short and uniform, as you have observed. We acknowledge the value in analyzing the overlaps between these responses to better understand the phenomenon of repeated answers. In our discussion of the impact of machine learning, we have now explicitly noted that the phenomenon of repeated responses was prominently confirmed in the results of this study. This is an important point that we will consider in our future research to provide more robust and nuanced analyses.

We also appreciate your insights on the discussion and conclusions being vague. As you rightly pointed out, additional research and potentially different methodologies are necessary to determine whether ChatGPT can apply ethical principles to diverse research scenarios and respond flexibly to subject protection opinions. 

However, a human being can consider a variety of ethical issues even when asked simple questions like those used in this study. Meanwhile, the interactive results using GPT-3.5 reveal many of the limitations of machine learning. We believe there is great value in documenting these in a scientific paper. We hope you understand this.

As I mentioned in the previous revision, this paper was submitted in April 2023, shortly after OpenAI released ChatGPT. Applying GPT-3.5 to research ethics is unprecedented, and we believe that this paper's appeal lies in explicitly examining various challenges and limitations in abstract questions like those in this study. We hope that this revised version and this response will be better understood.

Thank you again for your valuable feedback. We are committed to addressing these limitations in our ongoing and future research to develop more detailed and meaningful results and conclusions.

Attachment Submitted filename: Response to Reviewers.docx

# Peer review: Decision Letter 4

**DOI:** 10.1371/journal.pone.0311148.r009

**Authors:** Kwong Nui Sim

16 Sep 2024

Exploring Ethical Considerations in Medical Research: Harnessing Pre-Generated Transformers for AI-Powered Ethics Discussions

PONE-D-23-12275R4

Dear Dr. Mori,

We’re pleased to inform you that your manuscript has been judged scientifically suitable for publication and will be formally accepted for publication once it meets all outstanding technical requirements.

Within one week, you’ll receive an e-mail detailing the required amendments. When these have been addressed, you’ll receive a formal acceptance letter and your manuscript will be scheduled for publication.

An invoice will be generated when your article is formally accepted. Please note, if your institution has a publishing partnership with PLOS and your article meets the relevant criteria, all or part of your publication costs will be covered. Please make sure your user information is up-to-date by logging into Editorial Manager at Editorial Manager® and clicking the ‘Update My Information' link at the top of the page. If you have any questions relating to publication charges, please contact our Author Billing department directly at authorbilling@plos.org.

If your institution or institutions have a press office, please notify them about your upcoming paper to help maximize its impact. If they’ll be preparing press materials, please inform our press team as soon as possible -- no later than 48 hours after receiving the formal acceptance. Your manuscript will remain under strict press embargo until 2 pm Eastern Time on the date of publication. For more information, please contact onepress@plos.org.

Kind regards,

Kwong Nui Sim

Academic Editor

PLOS ONE

Additional Editor Comments (optional):

Reviewers' comments:

Reviewer's Responses to Questions

**Comments to the Author**

1. If the authors have adequately addressed your comments raised in a previous round of review and you feel that this manuscript is now acceptable for publication, you may indicate that here to bypass the “Comments to the Author” section, enter your conflict of interest statement in the “Confidential to Editor” section, and submit your "Accept" recommendation.

Reviewer #1: All comments have been addressed

Reviewer #9: All comments have been addressed

**********

2. Is the manuscript technically sound, and do the data support the conclusions?

The manuscript must describe a technically sound piece of scientific research with data that supports the conclusions. Experiments must have been conducted rigorously, with appropriate controls, replication, and sample sizes. The conclusions must be drawn appropriately based on the data presented. 

Reviewer #1: Yes

Reviewer #9: Partly

**********

3. Has the statistical analysis been performed appropriately and rigorously? 

Reviewer #1: N/A

Reviewer #9: N/A

**********

4. Have the authors made all data underlying the findings in their manuscript fully available?

The PLOS Data policy requires authors to make all data underlying the findings described in their manuscript fully available without restriction, with rare exception (please refer to the Data Availability Statement in the manuscript PDF file). The data should be provided as part of the manuscript or its supporting information, or deposited to a public repository. For example, in addition to summary statistics, the data points behind means, medians and variance measures should be available. If there are restrictions on publicly sharing data—e.g. participant privacy or use of data from a third party—those must be specified.

Reviewer #1: Yes

Reviewer #9: Yes

**********

5. Is the manuscript presented in an intelligible fashion and written in standard English?

PLOS ONE does not copyedit accepted manuscripts, so the language in submitted articles must be clear, correct, and unambiguous. Any typographical or grammatical errors should be corrected at revision, so please note any specific errors here.

Reviewer #1: Yes

Reviewer #9: No

**********

6. Review Comments to the Author

Please use the space provided to explain your answers to the questions above. You may also include additional comments for the author, including concerns about dual publication, research ethics, or publication ethics. (Please upload your review as an attachment if it exceeds 20,000 characters)

Reviewer #1: This is a new area of research that needs further exploration however, under the circumstances, the authors have done a fairly good job responding to the review comments despite the study limitations.

Reviewer #9: The ideea is interesting but a simple discussion on research ethics based on ChatGPT is very simplistit; could be more interesting to compare the approach the research based by ChatGPT with the same research based by specialists approach; the presentation should be much more complex

**********

7. PLOS authors have the option to publish the peer review history of their article (what does this mean?). If published, this will include your full peer review and any attached files.

If you choose “no”, your identity will remain anonymous but your review may still be made public.

**Do you want your identity to be public for this peer review?** For information about this choice, including consent withdrawal, please see our Privacy Policy.

Reviewer #1: No

Reviewer #9: No

**********

# Editor report: Acceptance letter

**DOI:** 10.1371/journal.pone.0311148.r010

**Authors:** Kwong Nui Sim

25 Sep 2024

PONE-D-23-12275R4 

PLOS ONE

Dear Dr. Mori, 

I'm pleased to inform you that your manuscript has been deemed suitable for publication in PLOS ONE. Congratulations! Your manuscript is now being handed over to our production team.

At this stage, our production department will prepare your paper for publication. This includes ensuring the following:

* All references, tables, and figures are properly cited

* All relevant supporting information is included in the manuscript submission,

* There are no issues that prevent the paper from being properly typeset

If revisions are needed, the production department will contact you directly to resolve them. If no revisions are needed, you will receive an email when the publication date has been set. At this time, we do not offer pre-publication proofs to authors during production of the accepted work. Please keep in mind that we are working through a large volume of accepted articles, so please give us a few weeks to review your paper and let you know the next and final steps. 

Lastly, if your institution or institutions have a press office, please let them know about your upcoming paper now to help maximize its impact. If they'll be preparing press materials, please inform our press team within the next 48 hours. Your manuscript will remain under strict press embargo until 2 pm Eastern Time on the date of publication. For more information, please contact onepress@plos.org.

If we can help with anything else, please email us at customercare@plos.org.

Thank you for submitting your work to PLOS ONE and supporting open access. 

Kind regards, 

PLOS ONE Editorial Office Staff

on behalf of

Dr. Kwong Nui Sim 

Academic Editor

PLOS ONE
